# From Heat to Electrons: Bridging Heterogeneous Liquid‐Phase Thermal and Electrocatalytic Oxidation of Ethylene Glycol over Co_3_O_4_

**DOI:** 10.1002/anie.202519188

**Published:** 2025-11-26

**Authors:** Catalina Leiva‐Leroy, Adarsh Koul, Falonne Bertholde Sharone Nkou, Jean Pascal Fandre, Akhil Hareendran, G. Wilma Busser, Harun Tüysüz, Stephane Kenmoe, Wolfgang Schuhmann, Martin Muhler

**Affiliations:** ^1^ Laboratory of Industrial Chemistry, Faculty of Chemistry and Biochemistry Ruhr University Bochum Universitätsstr. 150 D‐44780 Bochum Germany; ^2^ Analytical Chemistry – Center for Electrochemical Sciences (CES), Faculty of Chemistry and Biochemistry Ruhr University Bochum Universitätsstr. 150 D‐44780 Bochum Germany; ^3^ Department of Theoretical Chemistry University Duisburg‐Essen D‐45141 Essen Germany; ^4^ Heterogeneous Catalysis, Max‐Planck‐Institut für Kohlenforschung Mülheim an der Ruhr D‐45470 Germany; ^5^ IMDEA Materials Institute Calle Eric Kandel 2 Getafe Madrid 28906 Spain; ^6^ Max Planck Institute for Chemical Energy Conversion Mülheim D‐45470 Germany

**Keywords:** Ab initio molecular dynamics simulations, Cobalt oxide, Electrocatalysis, Ethylene glycol oxidation, Thermal catalysis

## Abstract

The selective oxidation of alcohols under thermal and electrocatalytic conditions presents a promising route to value‐added chemicals using sustainable energy sources. We establish mechanistic convergence between heterogeneous liquid‐phase thermal oxidation of ethylene glycol (EG) and its electrocatalytic oxidation reaction (EGOR) using mesostructured Co_3_O_4_ synthesized via hard templating as a catalyst. Comprehensive catalytic performance assessments and ab initio molecular dynamics simulations reveal analogous surface intermediates and pathways in both regimes, resulting in the same product distribution. Co^3+^ centers and OH^−^ species facilitate oxidation via proton‐coupled electron transfer (PCET), with molecular oxygen or the applied anodic potential regenerating active sites. Product selectivity to glycolate, formate, and oxalate is governed by temperature, EG concentration, pH, applied O_2_ pressure, or potential. Surface and bulk characterization confirm the robustness of the spinel structure, enabling multiple catalyst recycling. These insights provide a first mechanistic framework connecting heterogeneous thermal and electrocatalytic oxidation over non‐noble metal oxide catalysts, paving the way for designing multi‐functional materials for chemical synthesis under electrothermal conditions.

## Graphical abstract

The aerobic liquid‐phase thermal oxidation of ethylene glycol and its electrocatalytic oxidation resulted in the same product distribution using mesostructured Co_3_O_4_. Ab initio molecular dynamics simulations reveal analogous surface intermediates requiring Co^3+^ centers and OH^−^ species with O_2_ or the anodic potential regenerating the reduced active sites.

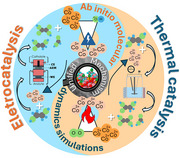

## Introduction

The selective oxidation of alcohols is a cornerstone of green chemistry, offering a route to high‐value chemicals such as aldehydes, ketones, and acids from renewable sources.^[^ ^1^ ^]^ Traditionally, industrial alcohol oxidation has relied on stoichiometric oxidants such as chromates, permanganates, or nitric acid, or on homogeneous catalytic systems using soluble metal salts with oxygen or hydrogen peroxide as the oxidants.^[^ ^2^, ^3^ ^]^ While effective, these methods suffer from serious drawbacks, including generating toxic waste, poor atom economy, harsh reaction conditions, and difficulties in catalyst recovery.^[^ ^4^ ^]^ In contrast, heterogeneous thermal catalysis offers a cleaner and more scalable solution with solid catalysts that enable continuous operation, efficient separation, and molecular oxygen as a benign oxidant.^[^ ^5^ ^]^ More recently, electrocatalysis has emerged as a promising alternative for alcohol oxidation, enabling milder temperatures with precise control via the applied potential and supported by the co‐production of green hydrogen at the cathode, suggesting a possible transition to an electrified and sustainable chemical industry.^[^ ^6^ ^]^

Integrating electrocatalysis with elevated temperatures and pressures could enable more efficient and greener processes by replacing thermal energy with the energy input via the applied cell voltage. To achieve this goal, a deeper mechanistic understanding is required as to whether similar adsorbates are formed on the catalyst surfaces across both regimes, leading to the same products with similar selectivity. And even more importantly, the question must be answered whether a single heterogeneous catalyst can operate efficiently with both thermal and electrochemical activation.

To explore these questions, alcohol oxidation serves as a useful probe reaction. It is feasible under heterogeneous thermal and electrocatalytic conditions, especially in alkaline media, ensuring comparable pH across systems. Among alcohols, ethylene glycol (EG) is an ideal candidate. It is a biogenic platform chemical, i.e., sustainably derived from, e.g., glycerol and offers low production costs and increasing industrial relevance.^[^ ^7^ ^]^ The selective oxidation of EG can yield glycolic acid (GA), formic acid (FA), and oxalic acid (OA) as products with diverse industrial applications. GA is the monomer for poly(GA) synthesis, a key biodegradable plastic.^[^ ^8^ ^]^ FA is attracting interest as a liquid organic hydrogen carrier (LOHC) due to its high hydrogen density.^[^ ^9^ ^]^ OA is widely used in pharmaceuticals, agriculture, and textiles.^[^ ^10^ ^]^ Therefore, enabling efficient and selective EG oxidation has strong potential in sustainable chemistry and energy applications.

Due to their excellent activity and selectivity, noble metal catalysts such as Pt, Pd, and Au have dominated the heterogeneous liquid‐phase thermocatalytic EG oxidation and the electrocatalytic EG oxidation reaction (EGOR).^[^ ^11^, ^12^, ^13^, ^14^, ^15^, ^16^, ^17^ ^]^ For instance, for heterogeneous thermal oxidation, Shi et al.^[^ ^12^ ^]^ achieved 100% EG conversion with 62% GA selectivity using Pt and Pt─Fe nanoparticles (NPs) on CeO_2_. Griffin et al.^[^ ^14^ ^]^ reported 90% EG conversion using Pd/C, Au/C, and PdAu/C catalysts. In the electrocatalytic domain, Marchionni et al.^[^ ^13^ ^]^ obtained 77.1% conversion and 55.5% GA selectivity in a direct EG fuel cell with a Pd‐(Ni─Zn)/C anode. Bellini et al.^[^ ^15^ ^]^ used a Rh complex on carbon in an organometallic fuel cell, achieving 38% conversion and 100% GA selectivity. Xin et al.^[^ ^11^ ^]^ demonstrated 100% EG conversion with Pt/C and Au/C in a DEGFC, with Pt reaching an GA yield of 76.1%. While these noble metals offer high performance, their scarcity and high cost limit industrial deployment.

Recent studies have turned to non‐noble catalysts, including NiSe_2_, Co‐Ni bimetallics, and iron‐based alloys, which have shown promising selectivity to glycolate and formate.^[^ ^18^, ^19^, ^20^, ^21^ ^]^ Among transition metal oxides, Co_3_O_4_ stands out due to its low cost, thermal stability, redox flexibility, and resistance to poisoning.^[^ ^22^, ^23^, ^24^ ^]^ The spinel structure of Co_3_O_4_ features dynamic Co^3+^/Co^2+^ redox transitions, which enable catalytic activity in both thermal and electrochemical settings. Additionally, hard‐templating strategies using mesoporous silica (e.g., SBA‐15) allow fine control over nanostructure, porosity, and surface area, possibly enhancing active site accessibility. Still, its performance and the mechanisms under heterogeneous thermocatalytic and EGOR conditions have not yet been systematically compared.

Recent conceptual advances are beginning to bridge the classical divide between heterogeneous thermal and electrochemical catalysis. Surendranath and coworkers^[^ ^25^ ^]^ demonstrated that net thermochemical aerobic oxidation reactions in water can be described as two electrochemical half‐reactions occurring on the catalyst surface. This framework has since been applied to proton‐coupled electron transfer (PCET) reactions at redox‐active interfaces, including nitroarene hydrogenation and hydroquinone oxidation.^[^ ^26^, ^27^, ^28^, ^29^ ^]^ Similarly, Adams et al.^[^ ^30^ ^]^ compared H_2_O_2_ formation during electrooxidation and gas‐phase thermal oxidation on noble metals, revealing convergent mechanisms. These studies collectively suggest that surface redox species act as the catalytically active sites. At the same time, external inputs primarily regenerate the active surface, be it temperature, oxygen pressure, pH, or electrochemical potential. However, this convergence of electro‐ and thermocatalytic mechanistic pathways has not yet been explored for EG oxidation using transition metal oxides as catalysts.

We show a comprehensive study of EG oxidation over a well‐defined mesostructured Co_3_O_4_ catalyst synthesized via hard templating. By systematically comparing the behavior of Co_3_O_4_ under heterogeneous thermal and electrocatalytic conditions, we evaluate performance metrics including conversion, selectivity, stability, and reusability, while elucidating surface mechanisms via ab initio simulations. Our findings reveal that Co_3_O_4_ is highly active and structurally stable under both regimes. More importantly, the Co^3+^ redox centers and OH^−^ species play analogous mechanistic roles in both systems. This allows us to propose a unified EG oxidation mechanism for Co_3_O_4_, driven by the Co^3+^/Co^2+^ redox cycle and OH^−^‐mediated surface oxygen transfer.

## Results and Discussion

### Heterogeneous Liquid‐Phase Thermocatalytic Oxidation

A series of kinetic experiments assessed the role of different experimental variables involved in the liquid‐phase thermal oxidation of EG over dispersed Co_3_O_4_ NPs, which were synthesized via the hard templating method using an SBA‐15 silica template according to a previously reported procedure (see additional results and discussion in the Supporting Information and Figures S1–S17; Tables S1–S5, therein).^[^ ^31^ ^]^ The effects of temperature, base, and O_2_ pressure on the conversion of EG and the resulting product distribution were investigated systematically. Table 1 shows the EG conversion after 6 h achieved with and without the addition of base. When the pH was neutral, almost no EG was converted (<1%). This observation correlates with the activity of other transition metals that showed no activity without OH^−^ ions in solution.^[^ ^32^ ^]^ Blank experiments were performed with KOH without a catalyst and exhibited similar low conversion and poor yield. Yuan et al.^[^ ^33^ ^]^ reported the use of a base acting as a homogeneous catalyst caused by easy proton abstraction from one of the hydroxyl groups, but higher conversion only occurred at significantly higher temperatures (300 °C).^[^ ^33^ ^]^ Hence, the catalyst was tested in the presence of KOH as a strong base and achieved a considerable yield of 17.5% FA with 29.5% EG conversion. Due to further oxidation, the selectivity of valuable products can be affected by, e.g., FA decomposition to CO_2_ and H_2_. CO_2_ further reacts with OH^−^ to form bicarbonate and carbonate, as has been reported for other transition metals.^[^ ^32^, ^34^ ^]^ Nevertheless, all experiments exhibited a carbon balance close to 100%, with small amounts of OA below the detection limit being present under milder reaction conditions. The high conversion of 29.5% achieved in the presence of both Co_3_O_4_ and KOH points to the high relevance of the deprotonation of EG, favoring its competitive adsorption on Co_3_O_4_ as ethylene glycolate. In addition, the K^+^ cations balance non‐covalent interactions between hydrated K^+^ and the hydroxylated Co_3_O_4_ surface, enhancing catalytic performance.^[^ ^35^ ^]^

**Table 1:** Effect of adding base on EG conversion and GA, FA, and OA yields at 120 °C and an O_2_ pressure of 10 bar.

Catalyst	Base	Ratio^a)^	Conversion (%)	Yield GA (%)	Yield FA (%)	Yield OA (%)
Co_3_O_4_	KOH	2	29.5	12.0	17.5	0
Co_3_O_4_	0	0	<1	<1	<1	0
No catalyst	KOH	2	3.3	2.8	<1	0

^a)^ Ratio of base:EG.

**Figure 1:**
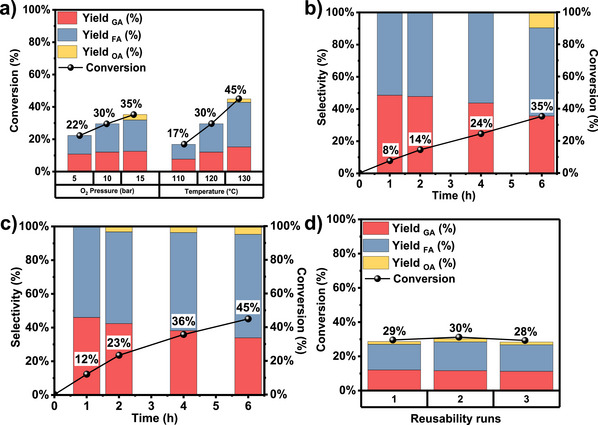
EG conversion over Co_3_O_4_ as a function of a) O_2_ pressure and temperature. Conversion and selectivity versus time profiles over mesostructured Co_3_O_4_ using a molar KOH:EG ratio of 2 at b) 120 °C and 15 bar O_2_ pressure and c) 130 °C and 10 bar O_2_ pressure. d) Reusability in EG oxidation for three consecutive oxidation runs.

Figure 1a shows conversion and product yields as a function of O_2_ pressure and temperature. Increasing conversion was observed for increasing O_2_ pressure up to 15 bar, demonstrating the critical role of O_2_ in the reoxidation of reduced Co^2+^ sites. As the O_2_ pressure increases, the higher dissolved O_2_ concentration enhances the rate of reoxidation of reduced Co^2+^ sites. However, no significant increase in the GA yield was observed, but rather the formation of higher oxidized products (Figure 1b). With increasing temperature, conversion improved, reaching 45% after 6 h at 130 °C. Even higher temperatures favored consecutive oxidation reactions yielding OA as C_2_ oxidation product and an enhanced yield of FA due to C─C bond cleavage in the intermediate GA (Figure 1c). Reusability studies were performed to determine the stability of Co_3_O_4_, demonstrating reproducible conversion and yields of GA, FA, and OA after a second and third run (Figure 1d). The high stability agrees with the structural robustness of the templated nanowires.^[^ ^31^ ^]^ Table S3 compares the mean sizes of the NPs and the crystallite sizes before and after reaction derived from TEM images and XRD results using the Scherrer equation. The XRD patterns originate from phase‐pure crystalline Co_3_O_4_ spinel. The lattice parameter was derived from the (311) diffraction peak of each Co_3_O_4_ sample before and after reaction and was essentially unchanged. The minor changes in mean NPs size and crystal size of Co_3_O_4_ in the 9 to 11 nm range are considered not significant.

Raman spectroscopy of the post‐reaction samples provides more insight into the structural changes during the reaction (Figures S3e,f and S13a). The Co_3_O_4_ catalyst shows the five vibration modes of the Co_3_O_4_ Raman spectrum before and after reaction using laser wavelengths of 785 and 532 nm. After the reaction, the modes were shifted to higher wavenumbers using the green laser excitation, indicating bond reordering due to partial reduction of Co^3+^ and possible oxygen vacancy formation in the near‐surface region.^[^ ^36^ ^]^ Defective structures also include the redistribution of cations, or in other words the occurrence of (partial) spinel inversion, in which Co^2+^ is moving into the octahedral voids.^[^ ^22^ ^]^ H_2_ temperature‐programmed reduction (TPR) was performed to observe changes in the oxidation state of the catalyst after reaction (Figure S16). After the reaction, a minor peak appeared at low temperatures of ∼100 °C, which can be attributed to active oxygen species adsorbed on oxygen vacancies.^[^ ^37^ ^]^ The catalyst showed no change in the Co^3+^ reduction temperature at 310 °C after reaction. Tables S2 and S4 summarize the quantitative changes in the H_2_ consumption for the two reduction peaks of the stepwise reduction Co^3+^→ Co^2+^→ Co^0^. The sample after reaction shows a Co^3+^ reduction peak, which is significantly lower in intensity compared with the fresh sample, pointing to a somewhat more reduced state after reaction in agreement with Raman spectroscopy. In summary, mesostructured Co_3_O_4_ synthesized by hard templating was found to be structurally resistant during thermal EG oxidation as confirmed by TEM, Raman spectroscopy, and XRD with no significant sintering and formation of by‐phases such as Co(OH)_2_.

### Electrocatalytic Ethylene Glycol Oxidation

**Figure 2:**
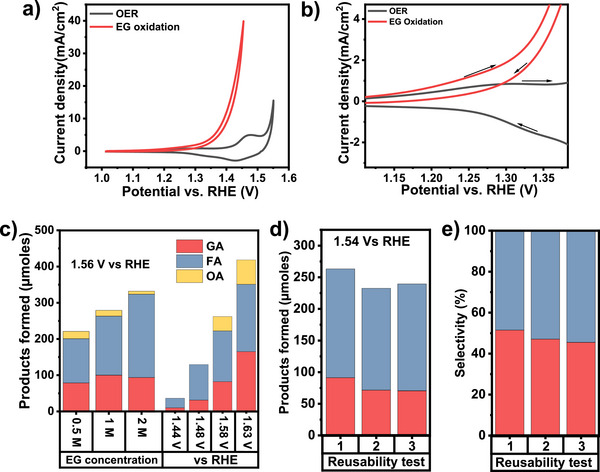
a) Cyclic voltammograms in 1 M KOH (OER) and 1 M KOH + 1 M EG (EGOR) on a Co_3_O_4_‐modified carbon paper electrode. b) Zoomed‐in view of the CV highlighting Co oxidation and EG oxidation currents. c) Product concentrations after chronoamperometry at different EG concentrations (0.5, 1, and 2 M) and different applied potentials (1.44, 1.48, 1.58, and 1.63 V versus RHE). d) Products formed and e) selectivity after three reusability tests of the catalyst at 1.4 V versus RHE.

Cyclic voltammetry (CV) in 1.0 M KOH (Figure 2a,b, black curve) using mesostructured Co_3_O_4_ reveals a prominent peak at 1.45 V versus RHE and a minor oxidation feature at 1.3 V versus RHE, typically attributed to the formation of surface oxides (Co^2+^ to Co^3+^ to Co^4+^).^[^ ^38^, ^39^ ^]^ Recent spectroscopic and theoretical studies suggested the involvement of non‐integer oxidation states (Co^2+δ^, Co^3+δ^), stabilized by hydroxide or oxide ligands.^[^ ^26^, ^40^ ^]^ Upon addition of EG, an increase in oxidation current is observed at potentials of about 1.3 V (Figure 2a,b, red curve), i.e., before the second redox wave, suggesting that EG oxidation is initiated during the first oxidation process of Co^2+^ to Co^3+^. The addition of EG also suppresses the Co redox peaks, indicating dynamic coupling between catalyst surface redox transitions and EG oxidation in agreement with a potential mechanism involving an electron‐transfer step with a coupled chemical follow‐up reaction. The CVs show the critical role of oxidized Co surface species in mediating both EG oxidation and the oxygen evolution reaction (OER).^[^ ^40^ ^]^

The effect of varying EG concentration (0.5–2 M) on conversion and selectivity was examined after 1 and 6 h of electrolysis in 1 M KOH, showing an expected enhanced current and total product formation with increasing EG concentration (Figures S6b and 2c). The products comprised a significant fraction of oxalate and formate at lower EG concentrations, while at higher EG levels overoxidation of initially formed oxidized species is suppressed and selective formation of glycolate and formate is promoted in good agreement with thermal EG oxidation. The product concentrations continued to rise after 6 h with an unchanged selectivity, especially for 2 M EG (Figure S7). Chronoamperometric measurements demonstrated increased catalytic currents with rising potentials from ∼8 mA cm^−2^ at 1.44 V to over 70 mA cm^−2^ at 1.63 V versus RHE (Figure S6c) accompanied by increased product formation from ∼50 µmol to over 450 µmol, respectively (Figure 2c). The product selectivity shifted from formate production at lower potentials to a more pronounced glycolate formation and emerging oxalate at potentials above 1.58 V, reflecting the activation of oxidation pathways avoiding C─C splitting (Figure S8a). Three consecutive 1 h reuse experiments were conducted at 1.53 V versus RHE to test the catalyst's longevity using new electrolyte for each cycle. Although a gradual decrease in initial current and glycolate yield was observed (Figures 2d and S6e), the results affirm the ability of the Co_3_O_4_ catalyst to recover active sites between cycles.

Extending the duration of the chronoamperometric electrolysis to 15 h at 1.54 V versus RHE revealed marked changes in activity (Figure S6d), product formation, and selectivity (Figure S8c). The current declined steadily, and the product profile shifted to oxalate as the major product (30%), while glycolate and formate decreased. With increasing product concentration over time, glycolate accumulated in the electrolyte, enhancing the probability of its re‐adsorption and oxidation, leading ultimately to higher concentrations of stable oxalate. A post‐run experiment in fresh electrolyte showed recovery of the initial selectivity, confirming that long‐term changes are not due to catalyst deactivation.

The catalyst's activity and stability were also studied after 6 h of heterogeneous thermal catalysis. Cyclic voltammograms and chronoamperometry (Figure S9) showed a slight decrease in the EG oxidation current density. CVs also revealed slightly higher current densities for the OER. The product formation also decreased (Figure S8b) from ∼280 to 240 µmol with a slight shift toward oxalate, while formate remained the dominant product. Electrochemical impedance spectroscopy (EIS) (Figure S10a) and the corresponding Nyquist plots revealed multiple overlapping semicircles, which were approximated by a three‐Randles element model assigned to surface relaxation (RCT1), CoOOH formation (RCT2), and EG oxidation (RCT3).^[^ ^41^, ^42^ ^]^ Distribution of relaxation times (DRT) analysis (Figure S10c) revealed three distinct time constants corresponding to these processes. At higher potentials, RCT1 diminished in intensity and contribution. RCT2 retained its characteristic time constant but decreased in resistance, suggesting facilitated Co oxidation. Most notably, RCT3 shifted to lower time constants and decreased in intensity at higher potentials, indicating accelerated EG kinetics. EG oxidation is enhanced at higher potentials, while earlier steps (surface oxidation) become less limiting or decoupled.

Classical work concerning electrocatalytic alcohol oxidation by Fleischmann et al.^[^ ^43^ ^]^ in the 1970s proposed a potential‐independent hydrogen atom transfer (HAT) mechanism involving the Ni(OH)_2_/NiOOH redox couple. Ni^2+^ is anodically oxidized to Ni^3+^, which then abstracts a hydrogen atom from the α‐carbon of a deprotonated alcohol (alkoxide), forming an aldehyde and regenerating Ni^2+^. Hence, the actual alcohol oxidation occurs via a chemical and potential‐independent step, though formation of Ni^3+^ is potential‐dependent. More recently, Bender et al.^[^ ^44^ ^]^ identified an alternative mechanistic pathway involving further oxidation of Ni to a higher‐valent Ni^4+^ species at more positive potentials. This Ni^4+^ species facilitates alcohol oxidation via a hydride transfer mechanism, i.e., inherently potential‐dependent. Bender et al.^[^ ^45^ ^]^ demonstrated that alcohols predominantly undergo a potential‐dependent hydride transfer, whereas aldehydes are predominantly oxidized via the potential‐independent HAT pathway. In either mechanistic route, the applied anodic potential regenerates the active site in a high oxidation state, which in turn oxidizes alcohol.

We propose, analogous to the Ni(OH)_2_/NiOOH system, that cobalt‐based oxyhydroxides, such as CoOOH, can also facilitate alcohol oxidation through a HAT‐type mechanism. At more positive potentials, further oxidation of the surface to Co^4+^ or CoO_2_‐like species may enable a hydride transfer pathway, in which the C─H bond cleavage and electron transfer occur concertedly. However, up to now no studies have shown the presence of Co^4+^ species during the oxidation of alcohols, suggesting that alcohol oxidation on cobalt oxides may proceed predominantly through a HAT mechanism rather than a hydride transfer pathway. However, discriminating between these mechanistic routes remains experimentally challenging, largely due to the overlapping redox processes in Co_3_O_4_ and the short‐lived nature of high‐valent Co^4+^ intermediates.

To transfer this knowledge to Co_3_O_4_‐catalyzed EGOR, we used in situ Raman spectroscopy (Figure S11). In the absence of EG and at low potentials (OCP to 1.4 V versus RHE), vibrational bands at ∼470, ∼520, and ∼690 cm^−1^ were observed, corresponding to the Eg, F_2g_, and A_1g_ modes of Co_3_O_4_ (Figure S13). At 1.5 V the A_1g_ band broadens and attenuates in agreement with surface oxidation. At potentials ≥1.6 V, a new band appears near 580–620 cm^−1^ attributed to the formation of CoOOH.^[^ ^46^ ^]^ Introducing EG to a CoOOH‐containing surface results in the immediate disappearance of the CoOOH Raman signature at open circuit conditions, and the subsequent application of an anodic potential ≥1.6 V does not regenerate the CoOOH signal (Figure S11c). Obviously, CoOOH is rapidly transformed upon reaction with EG. EG oxidation proceeds efficiently at these potentials, indicating that oxidized Co species such as surface‐confined Co^3+^/Co^4+^ or Co═O/Co─OH motifs, which are not Raman‐active, remain active.^[^ ^47^ ^]^ While the transient formation of H_2_O_2_ on Co_3_O_4_ under oxidizing alkaline conditions cannot be ruled out, its rapid decomposition renders it undetectable and prevents accumulation to measurable levels.^[^ ^48^ ^]^

Characterization of the post‐EGOR catalyst provides evidence for subtle surface and microstructural changes induced by prolonged electrolysis while preserving the bulk spinel structure. XPS (Figure S12b) shows a minor shift of the Co 2p peak to higher binding energy, consistent with a slight increase in the average cobalt oxidation state and/or a more covalent Co─O environment. This suggests an enrichment of oxidized cobalt motifs that remain catalytically active at anodic polarization in agreement with results from heterogeneous thermal oxidation. XRD patterns (Figure S13b) confirm the retention of the pure Co_3_O_4_ spinel phase. Rietveld refinement reveals a small yet measurable contraction of the lattice parameter from 8.120 to 8.109 Å, possibly due to the creation of oxygen vacancies. Raman spectroscopy of the post‐EGOR samples (Figure S14b) shows a slight shift of the Co─O bands to lower wavenumbers. This shift may be attributed to near‐surface defect formation, local bond weakening, or strain effects associated with electrochemical cycling and potential‐driven reconstruction.^[^ ^29^, ^30^ ^]^ In particular, oxygen vacancy creation and (partial) spinel inversion, where Co^2+^ redistributes to octahedral sites, can perturb the Co─O vibrational landscape and modulate Raman‐active modes, even if XPS suggests a higher average oxidation state at the surface. The higher Co 2p binding energy can be reconciled with softened Raman modes by considering the depth sensitivity of XPS, probing only the more oxidized outer shell, and of Raman spectroscopy, which samples a thicker layer in which defect‐mediated lattice relaxation and cation redistribution dominate the vibrational response. SEM (Figure S15) only reveals minor agglomeration and the appearance of micro‐cracks within the catalyst film after electrolysis, likely arising from local pH gradients and mechanical stress during chronoamperometric operation. Co_3_O_4_ preserves its crystallinity and functional redox responsiveness under EGOR conditions while undergoing moderate, largely reversible surface restructuring. These features are consistent with the catalyst's ability to sustain activity and selectivity over multiple electrochemical cycles, with performance changes related to interfacial phenomena rather than irreversible bulk degradation.

### Molecular Dynamics Simulations

Ab initio molecular dynamics simulations were used to investigate the facet‐dependent decomposition of EG on Co_3_O_4_ on the low‐index (001) and (110) facets. Heterogeneous thermal oxidation simulations were performed at 120 °C, and the impact of oxygen vapor pressure was addressed by considering 1 to 4 O_2_ molecules in the catalytic systems. To consider the dissociation energy of O_2_, 2 to 8 single atomic oxygen species adsorbed on neighboring surface Co sites were considered. This corresponds to O_2_:EG ratios of 1:4, 1:2, and 1:1, respectively. Alkaline conditions were created by considering KOH species solvated in a water film with a density of ∼1 g cm^−3^. The electrolyte was modeled considering a 2:1 KOH:EG ratio, i.e., 8 KOH molecules for 4 EG molecules. To assess the role of cations, a configuration without KOH and at the highest vapor pressure of an O_2_:EG ratio of 1:1 was considered. For electrochemical oxidation, room temperature simulations were performed. Besides the above‐mentioned alkaline conditions, an H‐depleted solvent was further considered, and the resulting hydroxyl moieties were employed as oxidizing agents within a 2:1 OH:EG ratio.

Considering the computational setup above, we monitored the decomposition of EG on the respective surfaces for a total simulation time of at least 20 ps in each case. Table 2 summarizes the intermediates, products, by‐products, and CoO_x_H_y_ species observed during the simulations. Co─OO, Co─OOO, and Co─OOH species were found on both surfaces, and hydrogen, oxygen, and hydroperoxide evolution are favored under almost all conditions. Co─OOO species are metastable and are the result of a dynamic interaction between two neighboring Co─OO and Co─O species. On the (001) surface, ethylene dioxy species, glycolaldehyde, formaldehyde, and hydroxy methyl groups can form. On the (110) surface, additional species that could be attributed to precursors of acetate or GA are present. On each surface, it is seen that the reactivity increases with the O_2_ pressure, and alkaline conditions are needed to go beyond 2e^−^ oxidation or the formation of C_1_ products. The adsorbed species interact with the surfaces via various binding modes and motifs. While monodentate covalent binding is the prominent mode on the (001), it coexists with the bidentate binding mode on the (110) surface (Figure S18): 1‐fold O_EG_ adsorption on a single Co, 2‐fold adsorption of O_EG_ on a single Co. Bidentate binding with 2‐fold adsorption of O_EG_ on two distinct Co are only observed in the absence of alkaline conditions. Alkali ions in solution interact with EG via one of its O_EG,_ hindering the bidentate adsorption on the surface.

**Table 2:** Products, intermediates, by‐products, and CoO_x_H_y_ species formed at different concentrations of O_2_ with and without KOH.

Oxidizing agents		Products	
n KOH	n O_2_	(001)	(110)
8	0	OHCH_2_CH_2_O^−^, H_2_, O_2_, Co─OO	OHCH_2_CH_2_O^−^, H_2_, O_2_, Co─OOH
8	1	OHCH_2_CH_2_O^−^, H_2_, O_2_	OHCH_2_CH_2_O^−^, H_2_, O_2_, Co─OOH
8	2	OHCH_2_CH_2_O^−^, H_2_, O_2,_ H_2_O_2_, Co─OOH	OHCH_2_CH_2_O^−^, H_2_, O_2_, Co─OO, formaldehyde, OHCH_2_‐O‐CH_2_O
8	4	OHCH_2_CH_2_O^−^, H_2_, O_2_, Co─OO, Co─OOO formaldehyde, hydroxy methyl group	OHCH_2_CH_2_O^−^, H_2_, O_2_, H_2_O_2_, glycolaldehyde, OCH_2_HCH_2_O_2_
0	4	OHCH_2_CH_2_O^−^, H_2_, O_2_, Co─OO, glycolaldehyde	OHCH_2_CH_2_O^−^, H_2_, O_2_, H_2_O_2_, glycolaldehyde, OCH_2_CHO

**Figure 3:**
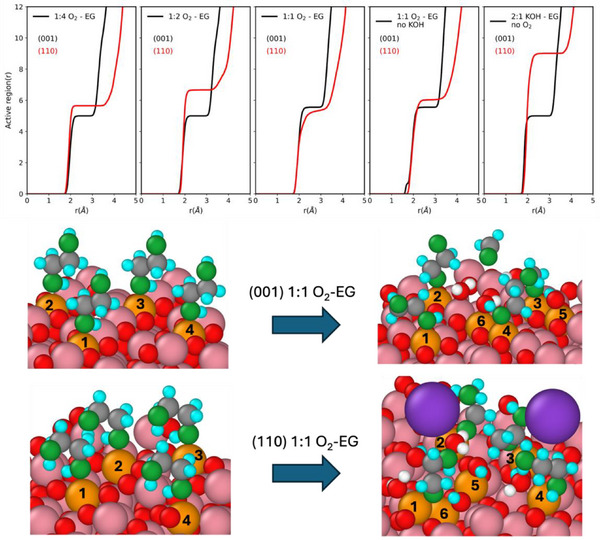
(Top) Renormalized running integrals of cobalt to EG's molecules RDFs displayed in Figure S18 for different concentrations of O_2_ with and without alkaline conditions. (Bottom) Expansion of active centers before and during the simulations. Co^3+^ are in pink, active Co^3+^ are denoted in gold, surface and water oxygens are in red, and water hydrogens are in white. The atoms of EG are colored: oxygen in green, carbon in grey, and hydrogen in cyan. EG can transfer a proton to surface oxygen and adsorbed oxygen.

To assess the size of the active regions, we calculated the coordination number of O_EG_ to surface Co, to which we added the number of neighboring Co bound to surface O, adsorbed atomic O, or OH, which act as proton or EG's H acceptors. The results exteriorize the fact that the active regions consist of several undercoordinated Co sites, which participate in stabilizing the adsorbed species. Figure 3 (top) shows that the active regions consist of 5 to more than 8 surface cobalt ions and that (110) displays larger active regions. Such behavior has already been reported in recent computational studies on the electrochemical oxidation of EG and 2‐propanol on the Co_3_O_4_ surfaces without alkaline conditions.^[^ ^49^, ^50^, ^51^, ^52^, ^53^, ^54^ ^]^

Figure 3 (bottom) shows examples of such collaborative active centers at work for the O_2_:EG = 1:1 ratio. Each Co to which EG molecules adsorb is counted as an active center (centers 1 to 4). On the (001) surface, two adsorbed atomic O serve as proton acceptors during the first decomposition step of one EG, leading to the formation of ethylene dioxy species (center 5) and during the formation of formaldehyde after the C─C bond breaking (center 6). On the (110) surface, besides one adsorbed atomic O that accepts a hydrogen from EG (center 5), one of the 3‐fold undercoordinated surface O receives a proton from EG and recovers its 4‐fold coordination (center 6). The EG molecule subsequently takes an OH^−^ present in the upper solvation shell. On each surface, the active regions consist of 6 undercoordinated Co atoms.

EG molecules compete with water to occupy the unsaturated Co sites on the surfaces. Water is found mainly in partially dissociated form at the interfaces, and both molecular water and hydroxide molecules are present. As illustrated in radial distribution functions of water O to surface Co (Figure S19), both OH and water molecules bind covalently on the surfaces via strong Co─OH and Co─H_2_O bonds as indicated by the peak region from 1.9 to 2.1 Å. One can distinguish between OH arising from deprotonation of intact molecules and surface OH forming upon proton transfer from molecular water to surface O^2−^. Water at the interface forms buckled layers of ∼ 3–4 Å thickness, as supported by the broadness of the O and H peaks in the density profiles reported in Figure S20 in the region from 13 to 16 Å and from 12 to 16 Å and on (001) and (110), respectively. Surface OH groups are represented by the peaks located at ∼1 Å below the lower limit of these regions. On both surfaces, the dissociation degree is high and increases with O_2_ concentration (Table S6).

**Figure 4:**
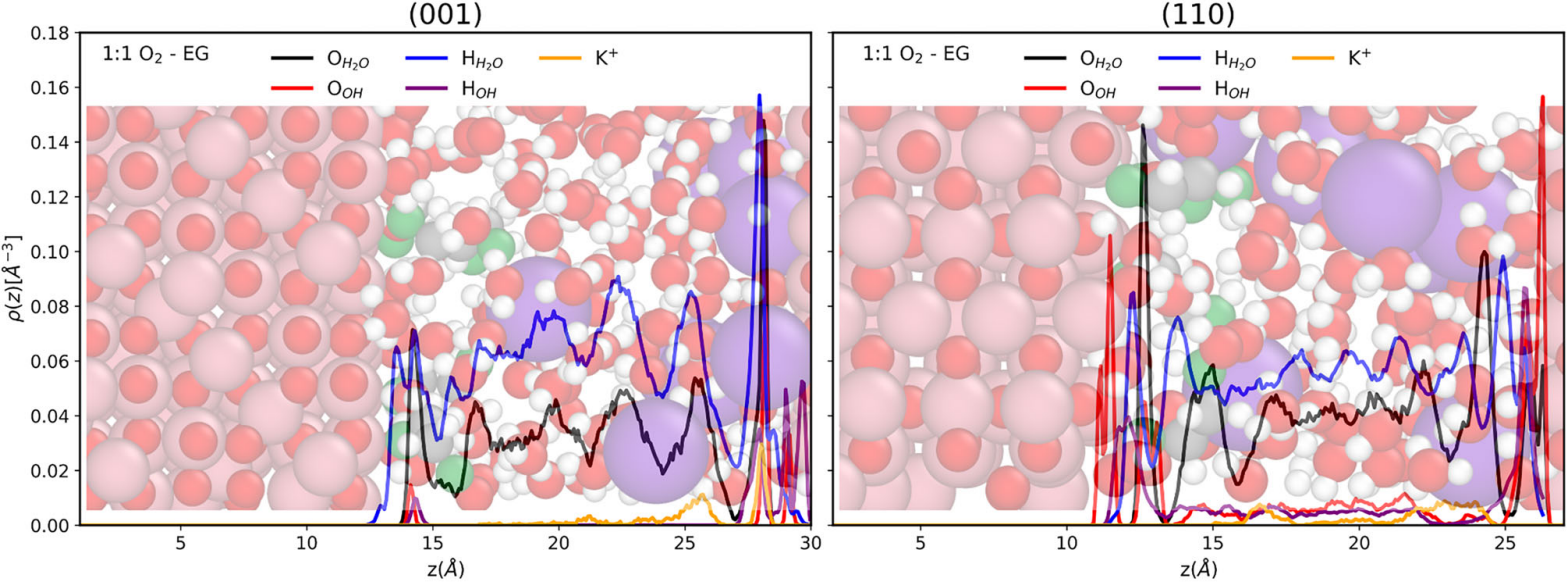
Figure 4. Average density profiles of H_2_O and hydroxyl's oxygen and hydrogen atoms and alkali ions at a 1:1 O_2_:EG ratio under alkaline conditions.

As mentioned above, alkaline conditions are needed to move toward 4e^−^ or obtain C_1_ oxidation products. Figure 4 illustrates the distribution of alkali ions concerning hydroxyls and molecular water at the highest oxygen concentration on both surfaces. It shows a more complex situation than commonly pictured in the literature, i.e., forming an electrochemical double layer between the alkali ions and adsorbed OH^−^. The alkali ions are placed initially into the electrolyte within a co‐planar distribution and reorganize into a depleted cationic layer in their final configurations. At least one out of the 8 K^+^ moves toward one of the half‐electrodes to interact with EG adsorbates within the electrolyte (regions from 18 to 20 Å in Figure 4) or even in the vicinity of the surface (region from 12 to 15 Å in Figure 4, right inset). At the same time, the rest diffuses toward the other half‐electrode (regions ∼30 Å in Figure 4). Both half‐electrodes are populated by H_2_O and OH^−^ molecules.

As can be seen from Figure S21, cationic layers are more ordered on the (001) surface. More diffused cationic layers are present on the (110) surface, with both adsorbed K^+^ coexisting with interfacial H_2_O and OH, and K^+^ located in the first solvation shells of bulk water. This yields the formation of (K‐OH‐H_2_O)_n_ complexes present both at the interfaces and in the electrolyte, and the first solvation shells of K^+^ that may include 5 to 6 hydroxyls or water molecules (Figure S22). Interestingly, the first coordination shell of (K‐OH‐H_2_O)n complexes may include EG's oxygens (see snapshot in Figure S22, bottom left). However, this does not imply that it always favors the decomposition of EG or the breaking of its C─C bond.

The reaction pathways of EG decomposition on the (001) and (110) surfaces are summarized in Figure S23 for the simulation time considered in this work. Under thermal oxidation and EGOR conditions, the reactivity increases with the amount of O_2_, and the (110) facet exhibits a higher reactivity. EG partial oxidation occurs on this facet first, as supported by forming 2e^−^ or C_1_ products at lower O_2_ content than the (001) surface. As indicated by the time distribution of intramolecular bond lengths in the OHCH_2_‐O‐CH_2_O intermediate observed on the (110) surface (Figure S24, middle inset, bottom), already at an O_2_:EG ratio of 1:2, C─C bond cleavage occurs around 2 ps (black curve), as indicated by the steep increase in the bond distance. This yields the formation of two formaldehyde groups. One HCHO group captures an OH^−^ from the electrolyte at 5 ps (see the decrease in the green curve) and further recombines with the second HCHO to form an epoxide ring, as indicated by a reduction in the orange curve. Meanwhile, the C═O double bonds in each HCHO moiety elongate with single‐bond character (blue and red curves).

In the absence of alkali cations (thermal oxidation), the reaction leads solely to the formation of C_2_ products, namely glycolaldehyde on the (001) surface and an acetal on the (110) surface. Under electrocatalytic conditions, room temperature simulations show the formation of single‐deprotonation intermediates, specifically ethylene dioxy species, on both surfaces. This can be attributed to the OH concentration in the water film considered in this work. Our recent studies in an alkaline‐free environment reported the formation of 2e^−^ products for twice the OH population and 4e^−^ products under more H‐deficient aqueous solvents.^[^ ^50^, ^51^ ^]^ So, the applied anodic potential tips the balance for electrocatalytic oxidation. However, in both catalytic environments, H_2_, O_2_, and H_2_O_2_ evolution and the formation of Co─OO, Co─OOH, and even metastable Co─OOO species are observed on the surfaces. In most cases, the surface‐assisted decomposition of EG proceeds via the Langmuir–Hinshelwood (LH) mechanism.

(1) $$\begin{array}{cc} & \mathrm{HO}-CH_{2}-CH_{2}-\mathrm{OH}\;+\mathrm{KOH}\;\\ & ⇌\mathrm{HO}-CH_{2}-CH_{2}-O^{-}+K^{+}+H_{2}O \end{array}$$

(2) $$\begin{array}{cc} & \mathrm{HO}-CH_{2}-CH_{2}-O^{-}+2\;Co^{3+}+OH^{-}\\ & \to \mathrm{HO}-CH_{2}-\mathrm{CHO}+2\;Co^{2+}+H_{2}O \end{array}$$

(3) $$\mathrm{HO}-CH_{2}-\mathrm{CHO}\;+OH^{-}⇌\mathrm{HO}-CH_{2}-\mathrm{CHO}\left(OH^{-}\right)$$

(4) $$\begin{array}{cc} & \mathrm{HO}-{\mathrm{CH}}_{2}-\mathrm{CHO}\left({\mathrm{OH}}^{-}\right)+2\;{\mathrm{Co}}^{3+}+{\mathrm{OH}}^{-}\;\\ & \to \mathrm{HO}-{\mathrm{CH}}_{2}-\mathrm{COOH}\;+2{\mathrm{Co}}^{2+}+H_{2}O \end{array}$$

(5) $$4\;Co^{2+}+O_{2}+2\;H_{2}O\to 4\;Co^{3+}+4\;OH^{-}$$

(6)

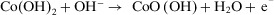




Integrating experimental and theoretical insights, we propose a unified mechanism for EG oxidation over Co_3_O_4_, i.e., operative under heterogeneous liquid‐phase thermal and electrocatalytic oxidation conditions [Equations ((1), (2), (3), (4), (5), (6))]. While the mechanism has been shown on one Co atom, the involvement of multiple (5–8) Co atoms for stabilizing the adsorbates is expected. The reaction begins with the deprotonation of EG by surface or interfacial OH^−^, followed by its adsorption on Co^3+^ centers [Equation (1)]. This step anchors the molecule and initiates a sequence of PCET events, during which EG undergoes oxidative dehydrogenation to yield intermediates such as glycolaldehyde [Equation (2)], and ultimately the C_2_ oxidation products glycolate and oxalate and the C_1_ product formate. Glycolaldehyde can easily form a geminal diol [Equation (3)], which is oxidatively dehydrogenated to GA [Equation (4)]. The Co^3+^/Co^2+^ redox couple acts as the catalytic engine in this transformation. In thermal oxidation, molecular oxygen serves as the terminal oxidant to reoxidize surface Co^2+^ back to Co^3+^ [Equation (5)]. In the EGOR, this regeneration is electrochemically driven by the applied anodic potential and OH^−^ [Equation (6)]. Despite the difference in the driving force, both regimes rely on the formation of similar surface motifs: strongly hydroxylated CoO_X_(OH)ᵧ layers with Co─OH and Co═O species, which actively mediate both oxidation and reoxidation steps. Ab initio molecular dynamics simulations confirm that the transformation from glycolaldehyde to formaldehyde occurs rapidly, especially under conditions favoring high oxygen activity or oxidizing potentials. This explains the frequent observation of formate and oxalate as dominant products under strongly oxidizing conditions. The simulations also reveal that EG interacts with multi‐site active regions composed of 5–8 Co atoms, some of which participate directly in adsorption while others stabilize adsorbed oxygen species or facilitate hydrogen/proton transfer. This dual‐platform insight offers a promising foundation for rationalizing next‐generation catalytic systems for selective polyol oxidation under combined thermal and anodic conditions.

## Conclusions

This study provides the first comprehensive mechanistic comparison of EG oxidation using mesostructured Co_3_O_4_ as a non‐noble heterogeneous catalyst under thermocatalytic and electrocatalytic conditions. Both heterogeneous thermal and electrocatalytic EG oxidation operate via convergent surface‐mediated mechanisms elucidated through a combination of thermal testing, electrochemical analysis, product selectivity profiling, post‐reaction characterization, and ab initio molecular dynamics simulations. In both systems, Co^3+^ motifs stabilized by OH^−^ serve as the active species, which are regenerated through molecular oxygen in thermal catalysis or the applied potential in the EGOR. Additionally, hydroxide ions facilitate the deprotonation of alcohols to generate reactive alkoxide species and assist further in oxidizing glycolaldehyde by forming a geminal diol. This finding suggests that heterogeneous thermal oxidation and electrocatalysis are fundamentally similar with respect to the surface redox chemistry, distinguished primarily by the applied driving force. Selectivity in both regimes is governed by reaction severity: lower potentials or milder thermal conditions favor partial oxidation products such as glycolate and formate, while more aggressive conditions promote overoxidation to oxalate and C─C bond cleavage to formate. High EG concentrations enhance selectivity toward C_2_ products by preferentially occupying active sites with EG. Post‐reaction characterization confirmed the structural robustness of Co_3_O_4_ under thermal and electrocatalytic conditions. While thermal catalysis induced slightly enhanced surface reducibility, electrocatalysis caused minor lattice contraction and surface oxidation without compromising catalyst integrity. In both cases, the spinel structure was preserved, and the catalytic performance remained stable over multiple reaction cycles. Co_3_O_4_ acts as a multi‐functional catalyst capable of mediating EG oxidation in electrochemical and thermal settings. The shared role of surface‐bound Co^3+^/Co^2+^ redox centers and OH^−^ intermediates underscores a unified catalytic mechanism resulting in the same product distribution, supporting the emerging view that thermal and electrochemical catalysis can be mechanistically bridged through surface redox chemistry.

## Supporting Information

The authors have cited additional references within the Supporting Information.

## Conflict of Interests

The authors declare no conflict of interest.

## Supporting information



Supporting Information

## Data Availability

The data that support the findings of this study are available in the Supporting Information of this article.
