# Supplementary material for: From Heat to Electrons: Bridging Heterogeneous Liquid‐Phase Thermal and Electrocatalytic Oxidation of Ethylene Glycol over Co3O4
Source: Angew Chem Int Ed Engl. 2025 Nov 26;65(3):e19188. doi: 10.1002/anie.202519188 (PMC12811663; doi:10.1002/anie.202519188)
Supplement: Supplementary file 1 — Supporting Information [file ANIE-65-e19188-s001.pdf]

Supporting information

From Heat to Electrons: Bridging Heterogeneous Liquid-Phase Thermal and Electrocatalytic Oxidation of Ethylene Glycol over Co_3O_4

Catalina Leiva-Leroy,[+] Adarsh Koul,[+] Falonne Nkou,[+] Jean Pascal Fandre, Akhil Hareendran, G. Wilma Busser, Harun Tüysüz, Stephane Kenmoe,* Wolfgang Schuhmann,* Martin Muhler*

[+] Catalina Leiva Leroy, Akhil Hareendran, Dr. G. Wilma Busser, [*] Prof. Dr. Martin Muhler
Laboratory of Industrial Chemistry; Faculty of Chemistry and Biochemistry; Ruhr University Bochum
Universitätsstr. 150; D-44780 Bochum, Germany
E-mail: martin.muhler@ruhr-uni-bochum.de

[+] Dr. Adarsh Koul, [*] Prof. Dr. Wolfgang Schuhmann
Analytical Chemistry – Center for Electrochemical Sciences (CES); Faculty of Chemistry and Biochemistry; Ruhr University Bochum
Universitätsstr. 150; D-44780 Bochum, Germany
E-mail: wolfgang.schuhmann@ruhr-uni-bochum.de

Falonne Nkou, [*] Dr. Stephane Kenmoe
Department of Theoretical Chemistry; University Duisburg-Essen
D-45141 Essen, Germany
E-Mail: stephane.kenmoe@uni-due.de

Jean Pascal Fandre, Prof. Dr. Harun Tüysüz
Heterogeneous Catalysis; Max-Planck-Institut für Kohlenforschung
D-45470 Mülheim, Germany
Email: tueysuez@kofo.mpg.de

Prof. Dr. Harun Tüysüz
IMDEA Materials Institute
Calle Eric Kandel 2, 28906 - Getafe, Madrid, Spain

Prof. Dr. Martin Muhler
Max-Planck Institute for Chemical Energy Conversion
D-45470 Mülheim, Germany

[+] These authors contributed equally to this work.

Experimental Section

Catalyst synthesis

SBA-15 silica was synthesized according to a reported procedure.[1] Ordered mesoporous Co_3O_4 was prepared via nanocasting using SBA-15 as the hard template through two-step impregnation. First, $Co(NO_3)_2 \cdot 6\,H_2O$ (Sigma-Aldrich, ACS reagent, >98%) was dissolved in pure ethanol to obtain a solution of 0.8 M. This precursor solution was impregnated into 1 g SBA-15 by first mixing 70 vol% of the solution with SBA-15 and stirring for 1 h at room temperature in a 250 ml PP beaker. The suspension was then dried at 40°C overnight, and the powder was calcined at 250°C for 4 h using 5°C min^{-1}. The calcined powder was then mixed with the rest of the 0.8 M solution for 1 h and dried again at 40°C overnight. A second calcination was performed at 250°C for 4 h with a heating ramp of 5°C min^{-1} and then further heating to 500°C at 2°C min^{-1}, which was held for 6 h. The SBA-15 hard template was leached out with 20 mL of 2 M NaOH at 70°C to ensure accessible silica removal. The mixture was decanted and the same solution was added and left overnight at 80°C. The dispersion was washed with DI water and then centrifuged. This was repeated until a neutral pH was obtained for the supernatant. The sediment was then dried overnight in a drying oven at 70°C, resulting in a mesostructured, crystalline Co_3O_4 nanowire network.

Powder catalyst characterization

N_2 adsorption-desorption isotherms were recorded at 77 K using a 3Flex Micromeritics set-up. Samples were degassed at 393 K for 4 h at 1.33 mbar. The specific surface area was derived according to the Brunauer–Emmett–Teller (BET) method in the 0.05 to 0.30 p/p_0 range. The pore size distribution was estimated by the Barrett–Joyner–Halenda (BJH) method applied to the desorption branch of the isotherm.

High-resolution (HR-TEM) and high-angle annular dark-field scanning transmission electron microscopy (HAADF-STEM) along with energy-dispersive X-ray (EDX) spectroscopy was performed using a Thermo Fisher Scientific Talos F200X transmission electron microscope operated at 200 kV. The software ImageJ was used to estimate the diameter of metal NPs. Frequency histograms of metal NPs sizes, mean size, and standard deviation have been calculated from more than 100 particles, where d_{pn} is the average particle size calculated (Eq. 1).

$$d_{pn} = \sum_i n_i d_i \,/\, \sum_i n_i \qquad (1)$$

X-ray diffraction (XRD) patterns were recorded using a Bruker D8 DISCOVER X-ray diffractometer, operating with Cu Kα radiation (λ = 0.15406 nm, 40 kV, 40 mA) and an energy-dispersive LYNXEYE XE-T detector. Data was collected within a 2θ range from 10° to 120°. The identification of phases and evaluation of crystal size was conducted using the software High Score Plus equipped with the Inorganic Crystal Structure Database (ICSD). The mean crystal size was derived using the Scherrer equation and the (311) reflection. All measurements were conducted at room temperature and atmospheric pressure.

H_2 TPR measurements were performed in a flow setup equipped with a stainless-steel U-tube reactor with a gas supply, a ceramic furnace for heating, and a thermal conductivity detector (TCD, Hydros 100). A certain amount of catalyst was pretreated in 50 sccm He (99.9999%) at 400 °C for 1 h. After cooling to 60 °C, the system was flushed with 50 sccm of 4.58% H_2 (99.9999%)/Ar (99.9995%) for 1 h. Then the setup was heated to 800 °C with a heating rate of 10 °C min^{-1} keeping the maximum temperature for 1 h.

Raman spectra were recorded using a LabRAM HR Evolution spectrometer (Horiba) equipped with a confocal microscope. The measurements were performed at room temperature using a green laser (Oxxius, 532 nm) at 100 mW and a near IR/red laser (IPS, 785nm) at 90 mW. 1% laser power was applied with 3 accumulations and integration for 600 s. The confocal point hole was set at 300 µm, and the samples were measured as powders on a microscope glass slide. A commercial Co_3O_4 (Merck) was used as reference; this was pre-treated at 600°C in synthetic air for 4 h to obtain a pure spinel structure.[2]

Methods

Thermocatalytic oxidation

The catalytic performance was evaluated in a batch reactor made of C-22 Hastelloy with a coated stirrer resistant to corrosion (Büchi). The standard reaction conditions were 50 mL aqueous solution of 0.325 M EG (anhydrous, 99.8%, Sigma−Aldrich), 0.65 M KOH (pellets, 99.99%, Sigma−Aldrich) to attain a pH ~13.68, and 50 mg of catalyst. The exact amounts added were 909 µL of EG and 1.84 g KOH. The autoclave was purged three times and then pressurized with 10 bar of O_2, followed by heating the mixture to 120 °C with a ramp of 5 °C min^{-1}. When the temperature of 120 °C was reached, mechanical stirring was turned on to start the reaction. Approximately 1−1.5 mL of samples were taken after 1, 2, 4, and 6 h and acidified with sulfuric acid for analysis by means of high-performance liquid chromatography (HPLC) (Azura, Knauer). An Aminex HPX-87H (Biorad) column was utilized to determine the concentrations of ethylene glycol, glycolic acid, formic acid, and oxalic acid.

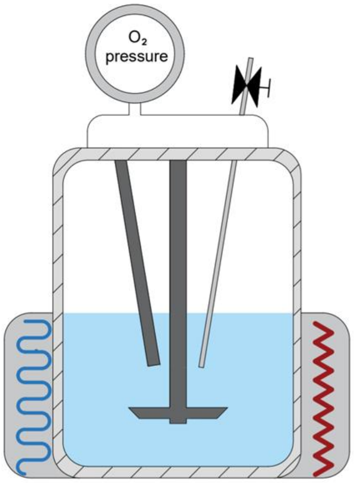

Figure S1. Scheme of the reactor for ethylene glycol thermal-liquid oxidation under pressurized aerobic conditions.

The conversion and selectivities were calculated using the response factor (RF). The reactant RF was set to 1 arbitrarily, and the RF of the products was calculated accordingly to the calibration curve as: $\mathrm{RF_{FA}} = \mathrm{RF_{EG}} \cdot \mathrm{Slope_{FA}}$ and $\mathrm{RF_{GA}} = \mathrm{RF_{EG}} \cdot \mathrm{Slope_{GA}}$.

The obtained response factors were used to calculate concentrations of the reactant and products as:

$$\mathrm{Concentration_{EG_f}(mmol\ L^{-1})} = \mathrm{Area_{EG_f}} \cdot \mathrm{RF_{EG}}$$

$$\mathrm{Concentration_{FA}(mmol\ L^{-1})} = \frac{1}{2}\mathrm{Area_{FA}} \cdot \mathrm{RF_{FA}}$$

$$\mathrm{Concentration_{GA}(mmol\ L^{-1})} = \mathrm{Area_{GA}} \cdot \mathrm{RF_{GA}}$$

$$\mathrm{Concentration_{OA}(mmol\ L^{-1})} = \mathrm{Area_{OA}} \cdot \mathrm{RF_{OA}}$$

Then selectivities and conversion were determined as:

$$\mathrm{Selectivity_i} = \frac{\mathrm{Concentration_i}}{\mathrm{Concentration_{EG_f}} + \mathrm{Concentration_{FA}} + \mathrm{Concentration_{GA}} + \mathrm{Concentration_{OA}}}$$

$$\mathrm{Conversion_{EG}} = \frac{\mathrm{Concentration_{FA}} + \mathrm{Concentration_{GA}} + \mathrm{Concentration_{OA}}}{\mathrm{Concentration_{EG_f}} + \mathrm{Concentration_{FA}} + \mathrm{Concentration_{GA}} + \mathrm{Concentration_{OA}}}$$

Reusability tests were conducted with the catalysts for three reaction runs under standard conditions. After each run, the catalysts were separated by centrifugation, washed three times with water, and dried overnight at room temperature. The missing amount of catalyst was adjusted with fresh catalyst for the subsequent run.

The reaction rate was determined as:

$$r_{EG}\,(\mathrm{mmol\ L^{-1}\ h^{-1}}) = c_{EG,0} \cdot \int_0^1 \left(\frac{dX}{dt}\right) \quad (2)$$

The slope was obtained from the plot of EG conversion versus reaction time. $c_{EG,0}$ refers to the initial concentration of EG.

Electrocatalytic oxidation

Flow-through cell setup

Electrochemical measurements were performed in a custom-designed flow-through cell (FTC) composed of two compartments separated by a Fumasep FAA-3PK-130 anion exchange membrane. The anode and cathode compartments were connected to separate electrolyte reservoirs (anolyte and catholyte), which were circulated at a constant flow rate of 12 mL min^{-1} using peristaltic pumps. A Ag/AgCl (3 M KCl) electrode served as the reference, and a Ni mesh was used as counter electrode. The working electrode was a Co_3O_4 carbon paper modified with the catalyst by drop coating.

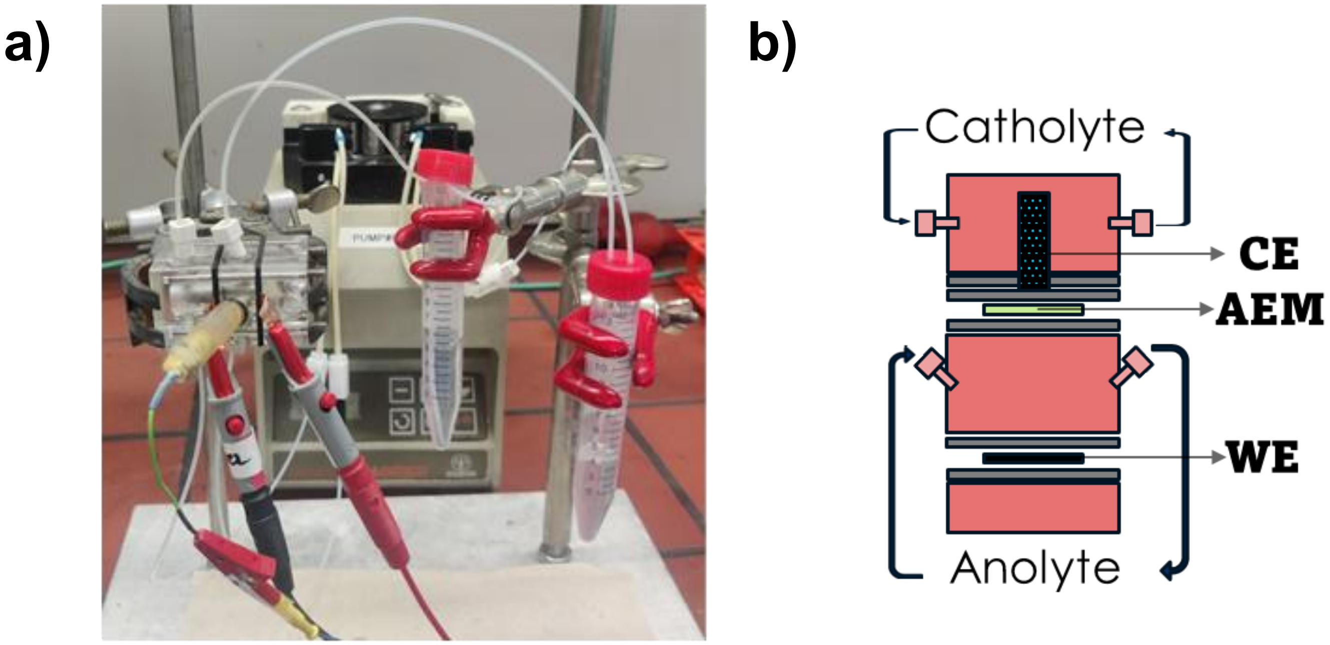

Figure S2. (a) Photograph of the flow-through cell setup used for electrochemical measurements. The setup includes electrolyte reservoirs, peristaltic pumps, and electrode electrical connections. (b) Schematic diagram of the flow cell configuration showing the catholyte and anolyte compartments having the Counter Electrode (CE) and Working electrode (WE) respectively, separated by an anion exchange membrane (AEM).

Working electrode preparation

The mesostructured Co_3O_4 catalyst was deposited onto an H23C2 Freudenberg carbon paper electrode via drop-casting. A catalyst loading of 1 mg/cm^2 was used for all measurements. Electrodes were dried at room temperature. A vacuum pump was used during the drop-casting process to accelerate and ensure uniform drying of the catalyst layer.

Cyclic voltammetry (CV) and linear sweep voltammetry (LSV)

Electrochemical testing was conducted in a three-electrode setup using 1 M KOH as the supporting electrolyte. The working electrode was modified with the hard-templated Co_3O_4 catalyst, and a Pt counter electrode and an Ag/AgCl (3 M KCl) reference electrode complemented the 3-electrode setup. Each measurement sequence consisted of a) open circuit potential (OCP) stabilization for 100 s to equilibrate the electrode-electrolyte interface, b) electrochemical impedance spectroscopy (EIS) in the frequency range from 100 kHz–1 Hz with a 10 mV$_{pp}$ AC amplitude to determine

the uncompensated solution resistance (R_u), which was used for iR compensation, c) cyclic voltammograms (CV) in the potential range from 0.0 to 0.6 V vs Ag/AgCl (3 M KCl) at a scan rate of 10 mV s^{-1}. The forward scan of the second cycle was extracted as linear sweep voltammogram (LSV). After this, 1 M ethylene glycol (EG) solution was added to the electrolyte, and the same sequence - OCP, EIS, and CV - was repeated under reaction conditions. Finally, chrono-amperometry (CA) was conducted at specific applied potentials (e.g., 1.44–1.63 V vs RHE) to evaluate catalytic activity, stability, and product evolution under steady-state operation.

Conversion of potentials to the RHE scale and iR compensation

All measured potentials were converted to the reversible hydrogen electrode (RHE) scale using the following equation:

$$E_{RHE} = E_{Ag/AgCl/\ 3\ M\ KCl} + E^{o}_{Ag/AgCl/3\ M\ KCl} + 0.059 \times pH$$

Where $E_{Ag/AgCl/\ 3\ M\ KCl}$ is the measured potential versus the reference electrode, and $E^{o}_{Ag/AgCl/3\ M\ KCl}$ = 0.207 V at 25 °C. The pH of the solution was estimated using:

$$pH = 14 + \log(c(OH-)) + \log(\gamma)$$

The activity of water (γ) was assumed to be 0.766, consistent with literature values; All conversions assumed measurements were taken at room temperature (25 °C).

To correct for ohmic drop (iR compensation), the following equation was applied:

$$E_{RHE,\ corrected} = E_{RHE} - i \times R_u$$

Where i is the current obtained from the voltammogram, and R_u is the uncompensated solution resistance obtained from EIS measurements. iR correction was applied to all CV and LSV data, but not to chronoamperometry (CA) or EIS-based charge transfer resistance measurements.

Electrochemical impedance spectroscopy (EIS)

EIS measurements were carried out using a frequency response analyser in a frequency range from 100 kHz to 1 Hz with a sinusoidal AC amplitude of 10 mV_{pp}.

- Solution resistance (Ru):

Measured at open-circuit potential (OCP). R_u was taken as the real part of the impedance at the highest frequency (100 kHz).

- Charge transfer resistance (R_{ct}):

Determined at applied potentials of 0.45, 0.55, 0.65, and 0.75 V vs. Ag/AgCl (3 M KCl) in a frequency range from 100 kHz to 10 Hz

- Equivalent circuit fitting and distribution of relaxation times (DRT) analysis

EIS data were processed and fitted using the RelaxIS 3 software. DRT was calculated using RelaxIS 3 applying a Gaussian function, a discretization factor of 10^{-5}, and the second derivative of the radial basis function (RBF).

Product quantification

For product analysis, 500 µL of the reaction mixture was collected and acidified with an equal volume of 0.53 M sulfuric acid. Product quantification was based on the number of micromoles of liquid products formed. Conversion was calculated as the total micromoles of detectable products, and selectivity was expressed as the fraction of individual products relative to the total amount of liquid products formed. Like thermal catalysis, high-performance liquid chromatography (HPLC) was used to detect and quantify products.

Electrode characterization

Surface morphology was characterized by SEM using a Quanta 3D FEG scanning electron microscope (FEI) operated at 30.0 kV. Crystallographic information was obtained from powder X-ray diffraction measurements performed on a Bruker D8 Discover diffractometer employing Cu Kα radiation (λ = 1.5418 Å) over a 2θ range of 5° to 90°. Surface

elemental composition and chemical states were examined via X-ray photoelectron spectroscopy (XPS) using an AXIS Nova system (Kratos Analytical) equipped with a monochromatic Al Kα X-ray source (1487 eV, 15 mA). The system was operated under ultra-high vacuum (~2×10^{-9} mbar), and high-resolution scans were acquired at a pass energy of 20 eV. A flood gun was applied for charge compensation, and spectra were calibrated using the C 1s peak at 284.8 eV. Raman spectroscopy was conducted on a LabRAM HR system (Horiba Jobin Yvon HR550) utilizing a 532 nm excitation laser, a water immersion objective (Olympus LUMFL, 60×, NA = 1.10), a monochromator with 1800 grooves/mm grating, and a Synapse CCD detector.

Results

Catalyst synthesis and characterization

Table S1 summarizes the textural properties of the mesostructured Co_3O_4 spinel synthesized by hard templating. The BET specific surface area was 122 $m^2 g^{-1}$ and showed a type IV(a) isotherm with the characteristic hysteresis loop of a mesoporous catalyst (Figure S3a). The sample presented an average pore size of 8.4 nm and pore volume of 0.3 $cm^3 g^{-1}$. These results are attributed to the lower pore sizes in the nanoparticles observed for the distribution around 3 nm in accordance with the wall thickness of the SBA-15 template.[3] Assuming spherical particles, the mean sizes were derived from the TEM images, and using the Scherrer equation the mean crystallite size was calculated (Table S1). Similar results were obtained for both measurements within the error deviation. Furthermore, the metal oxide particle size distributions were symmetric, and the histogram showed a Gaussian distribution (Figure S4).

The XRD pattern for the Co_3O_4 sample is displayed in Figure S3b. Results showed distinct reflections of the pure Co_3O_4 spinel phase (ICSD: 98-002-4210). The crystallite size was derived from the width of the (311) diffraction peaks by applying the Scherrer equation. Additionally, from this diffraction peak, the crystal structure exhibited a lattice parameter of 8.07 Å, suggesting constraining or defects present in the structure compared to the reported value for Co_3O_4 spinel of 8.08 Å.[4] The HRTEM images enable the determination of the lattice spacings of the cobalt spinel NPs. Figure S3c shows the presence of aligned Co_3O_4 nanowires, as reported previously.[5] The sample showed (311) and (111) lattice spacing at 0.243 and 0.462 nm, respectively.[6] The larger (111) lattice spacing may be due to a higher content of Co^{2+} ions and oxygen vacancies.[7] This can also be beneficial as products may rapidly migrate into and out of the structure.[8]

H_2 TPR experiments studied the reducibility of the samples (Figure S3d). The TPR profile show a first peak at 310 °C due to the reduction of Co^{3+} ($Co_3O_4 + H_2 \rightarrow 3CoO + H_2O$) and a second peak at 447 °C for Co^{2+} ($3CoO + 3H_2 \rightarrow 3Co + 3H_2O$).[9] It has been reported that lower Co^{3+} reduction temperatures are correlated with enhanced oxygen mobility and reactivity.[10] Table S2 shows the resulting hydrogen consumption for the first and second reduction of the Co_3O_4 samples. The H_2 uptake of the first reduction peak was quantified by integration to the minimum between the two TPR peaks.[11] A slightly higher amount of Co^{3+} is present in the bulk structure of the sample according to the Co^{3+}/Co^{2+} ratio.[12]

Raman spectroscopy was applied using different wavelengths of 532 and 785 nm, as well as confocal point holes (Figures S3e, S3f). The near-infrared laser (785 nm) delivers information mainly from the bulk of the material. The green laser (532 nm) provides information from the near surface region. Both lasers showed the five characteristic Co_3O_4 Raman modes F_{2g} (1), E_g, F_{2g} (2), F_{2g} (3), and A_{1g} .[13] No additional bands were observed, indicating that the bulk of the material consists of pristine Co_3O_4, due to the synthesis method of templated nanowires being highly robust and leading to phase-pure and stable polycrystalline Co_3O_4 catalysts.[3] For the 532 nm laser, the relative intensities of the F_{2g} (1) and the F_{2g} (2) bands were different compared to those obtained with the 785 nm laser. The position of the A_{1g} band indicates the presence of defects and/or differences in primary particle size in the nanometre range.[14] No significant redshift was observed with respect to the crystalline bulk material.

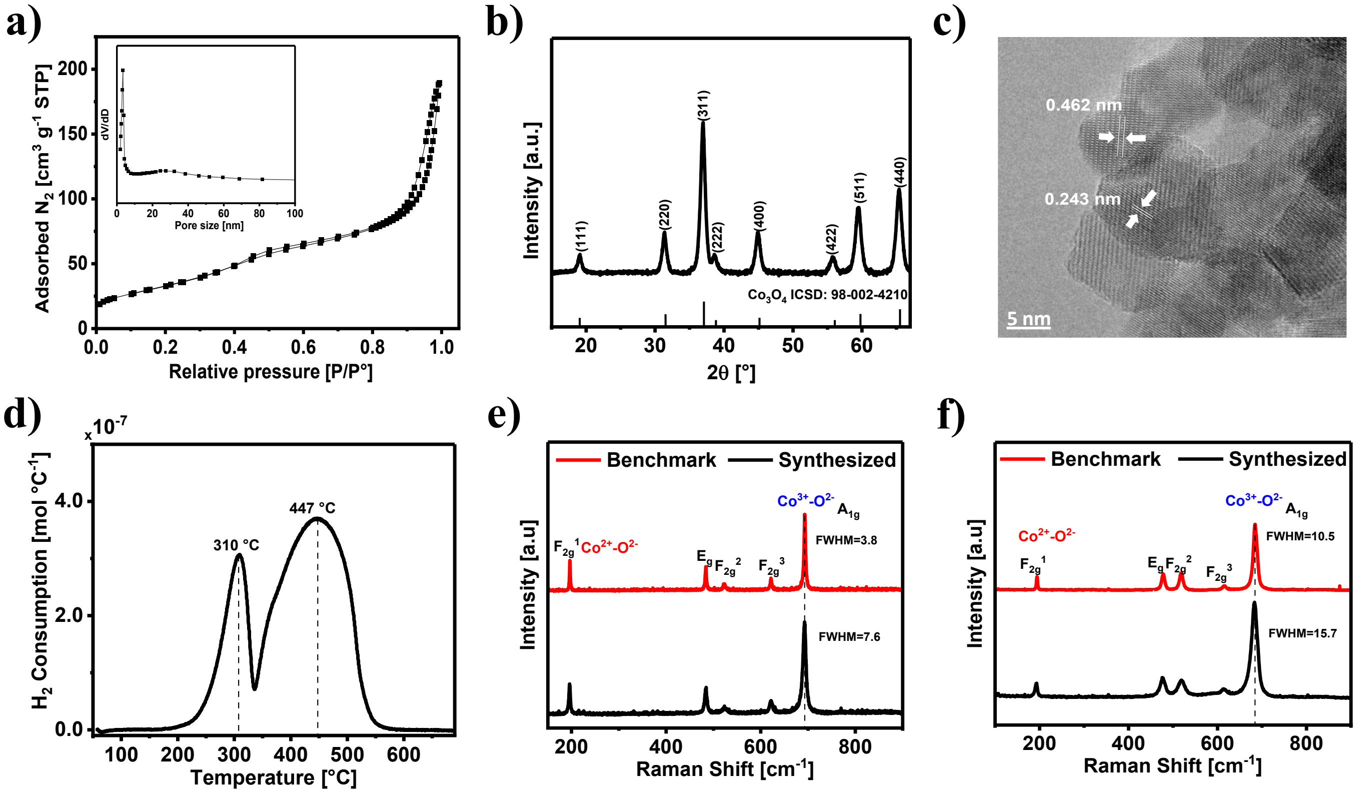

Figure S3. a) N_2 adsorption–desorption isotherms at 77 K and pore size distribution. b) XRD patterns of the Co_3O_4 spinel. c) HRTEM image. d) H_2-TPR profiles of Co_3O_4. Raman spectra of the commercial Co_3O_4 and the mesostructured samples using a confocal point hole of 300 μm with e) the 785 nm and f) the 532 nm laser.

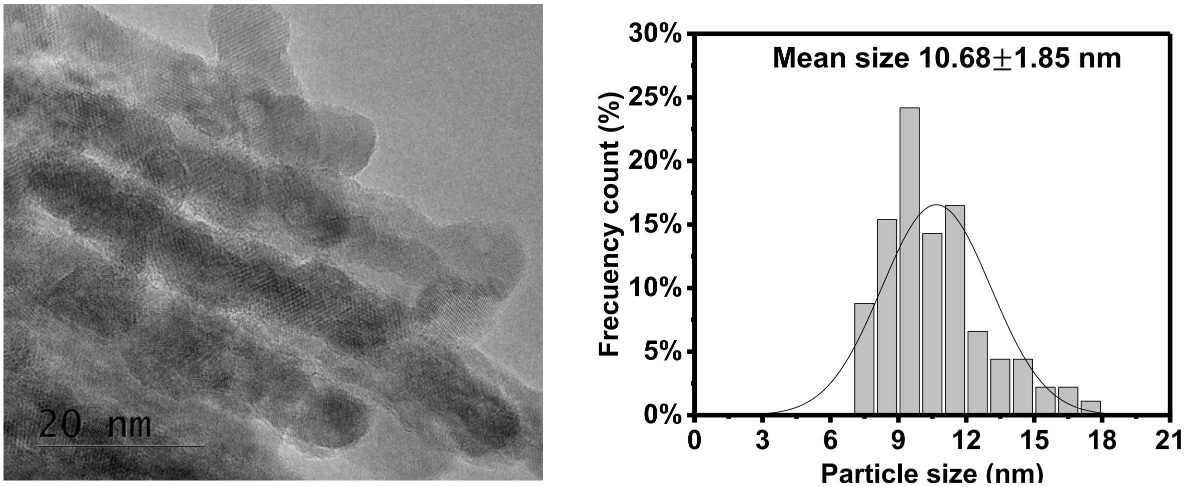

Figure S4. TEM image and particle size distribution.

Table S1. Summary of TEM, XRD, and N_2 physisorption results.

Catalyst	TEM	XRD	BET/BJH analysis		
	d_P[a] (nm)	d_P[b] (nm)	S_{BET} (m^2/g)	Pore size (nm)	Pore volume (cm^3/g)
Mesostructured Co_3O_4	10.7	8.7	122	8.4	0.300

[a] Calculated by the number average using 100 nanoparticles.

[b] Calculated using the Scherrer equation.

Table S2. Hydrogen consumption before reaction during the reduction of Co_3O_4 to metallic Co^0.

	**$Co^{3+} \rightarrow Co^{2+}$**	**$Co^{2+} \rightarrow Co^0$**	
**Mass (mg)**	**1st H_2 consumption (mmol)**	**2nd H_2 consumption (mmol)**	$\frac{Co^{3+}}{Co^{2+}}$
50.4	0.18	0.50	2.2

Thermocatalytic oxidation

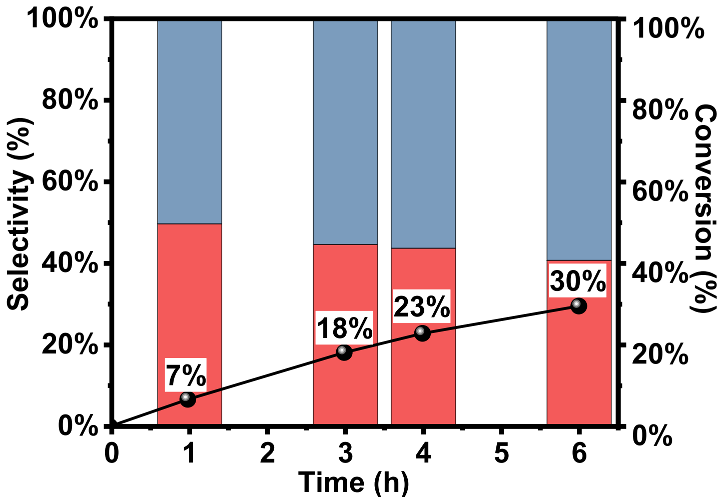

Figure S5. Conversion and selectivity vs. time profiles over Co_3O_4 in the liquid phase oxidation of EG at standard conditions at 120 °C and 10 bar O_2 pressure using a molar KOH: EG ratio of 2. GA FA

Electrocatalytic oxidation

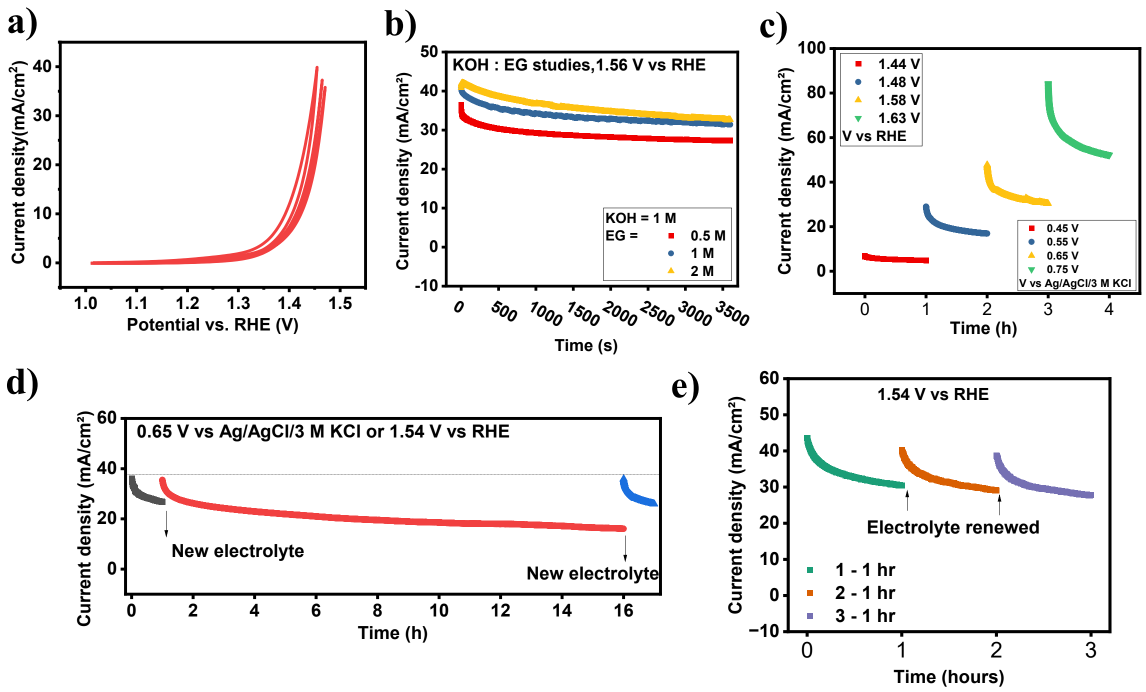

Figure S6. a) Three consecutive CVs in 1 M KOH and 1 M EG. Chronoamperometry measurements b) at 1.56 V vs RHE in varying EG concentrations (0.5 M,1 M, 2 M) and 1 M KOH, c) at applied potentials of 0.45 V, 0.55 V, 0.65 V, 0.75 V vs Ag/AgCl/3 M KCl in 1 M EG and 1 M KOH, d) long-term electrolysis (15 h) at 1.54 V vs RHE, preceded and followed by 1 h electrolysis at the same potential, e) at 1.54 V vs RHE for three reusability tests where the electrolyte was refreshed after each hour of electrolysis.

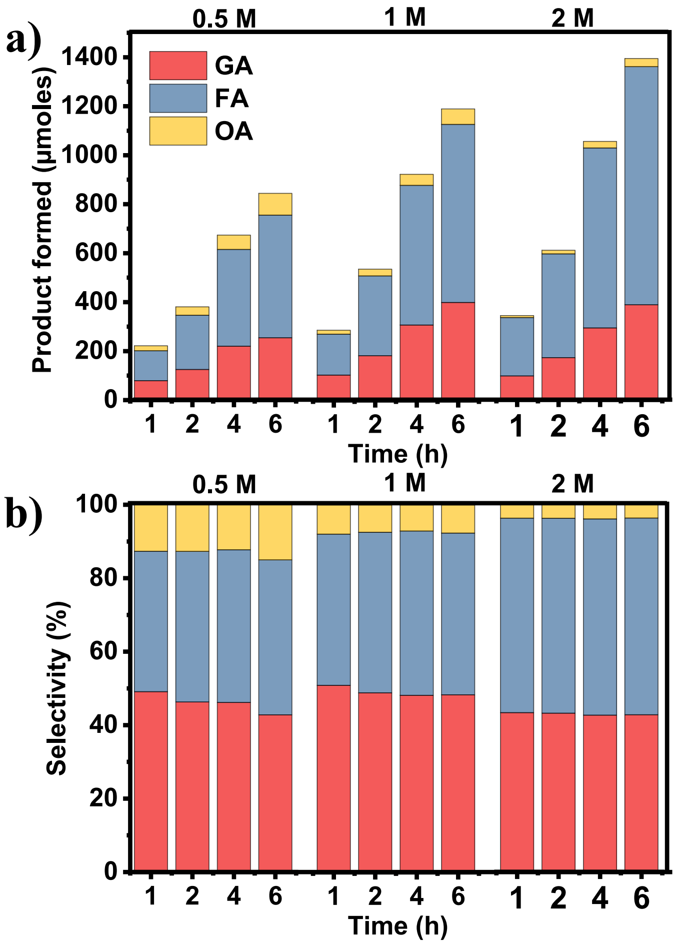

Figure S7. a) Conversion and b) selectivity: EGOR products - glycolate, formate, and oxalate - at varying EG concentrations (0.5 M, 1 M, and 2 M) during different reaction times (1 h, 2 h, 4 h, and 6 h) performed at 1.56 V vs RHE.

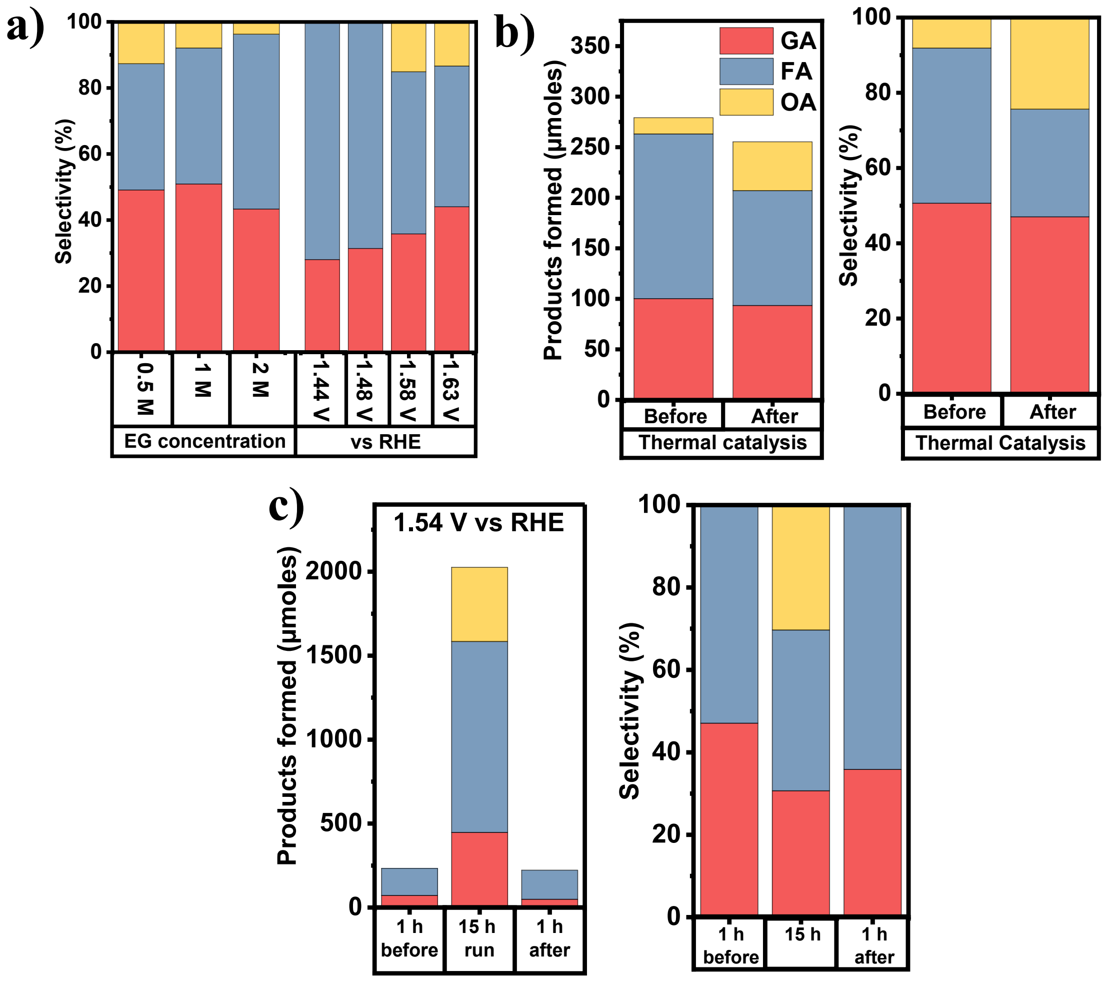

Figure S8. a) Product selectivity after chronoamperometry at different EG concentrations (0.5 M, 1 M, 2 M and 1 M KOH) at 1.56 V vs RHE, and different applied potentials (0.45 V, 0.55 V, 0.65 V, 0.75 V, vs Ag/AgCl/3 M KCl in 1 M EG and 1 M KOH. b). Reusability tests at 1.54 V vs RHE, 1.56 V vs RHE on pre- and post-thermal-treated catalyst. c) Products formed and selectivity distribution during long-term (15 h) electrolysis at 1.54 V vs RHE, along with catalytic results before and after 1 h electrolysis GA FA OA

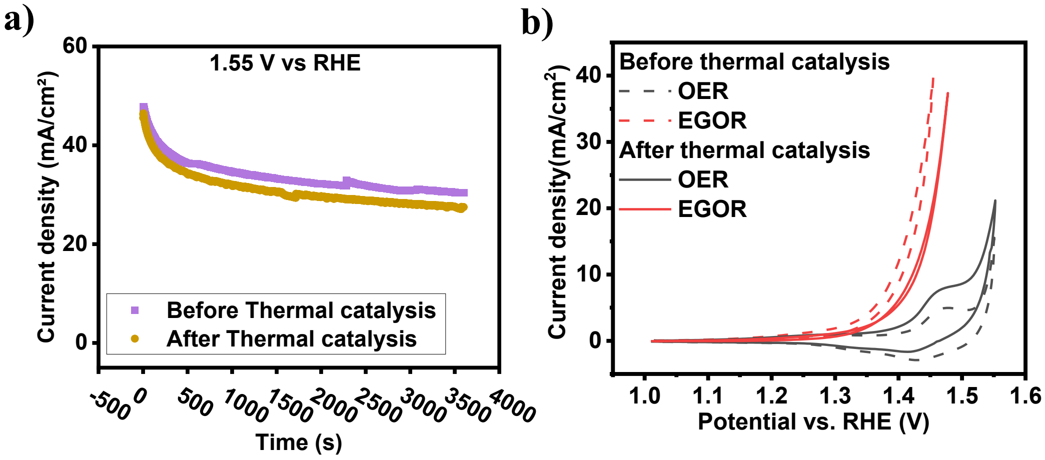

Figure S9. a) Chronoamperometry and b) cyclic voltammograms (OER and EG oxidation) on Co_3O_4 before and after thermal catalysis.

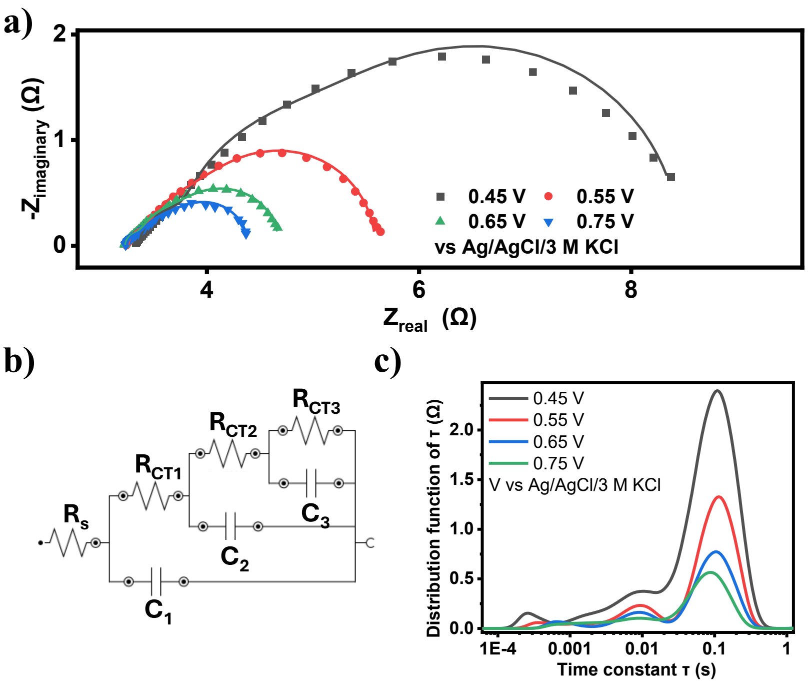

Figure S10. (a) EIS Nyquist plots at various applied DC potentials (0.45 V to 0.75 V vs Ag/AgCl/3 M KCl) (resembling the chronoamperometric measurements at different potentials), showing a decreasing semicircle diameter with increasing potential indicating changes in charge transfer resistance. (b) Equivalent electrical circuit model used to fit the impedance data, consisting of multiple R-CPE elements in series to represent the different electrochemical processes. (c) Distribution of relaxation times (DRT) analysis showing distinct peaks corresponding to different time constants, with potential-dependent shifts suggesting variation in interfacial processes and kinetics.

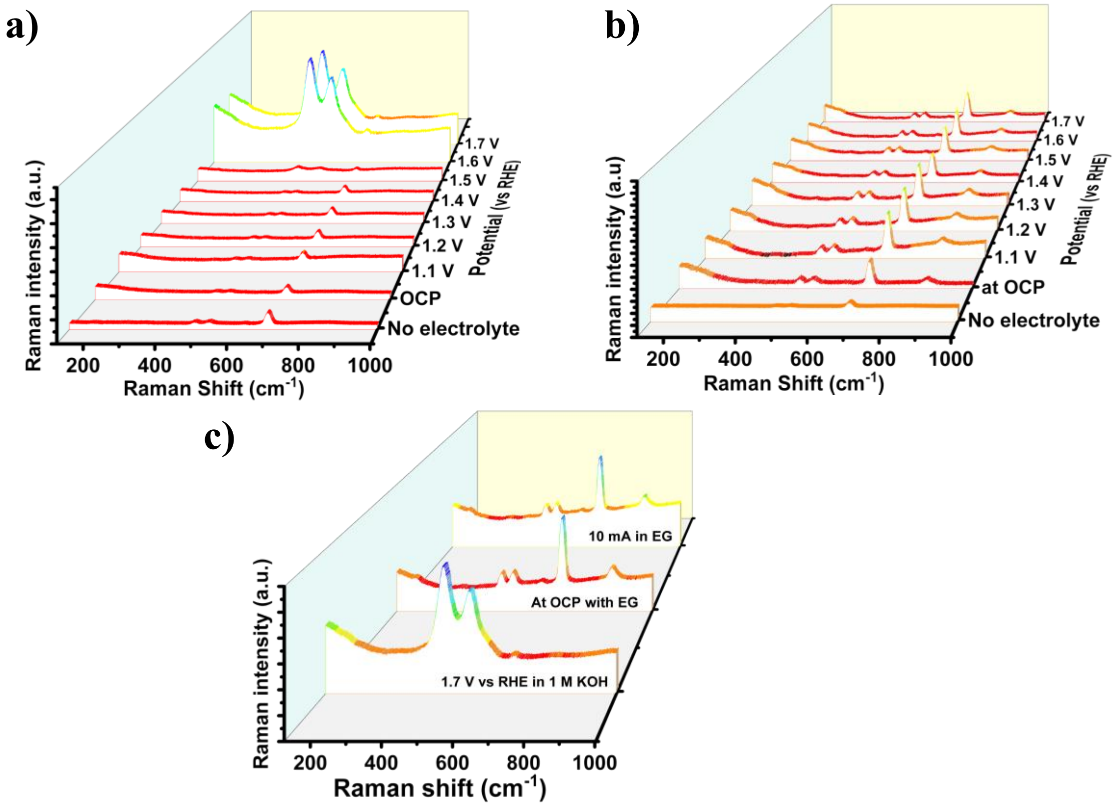

Figure S11. In-situ Raman spectroscopy a) in 1 M KOH (OER), b) 1 M EG + 1 M KOH (EG oxidation), c) with addition of EG without applying potentials.

Post-reaction characterization results

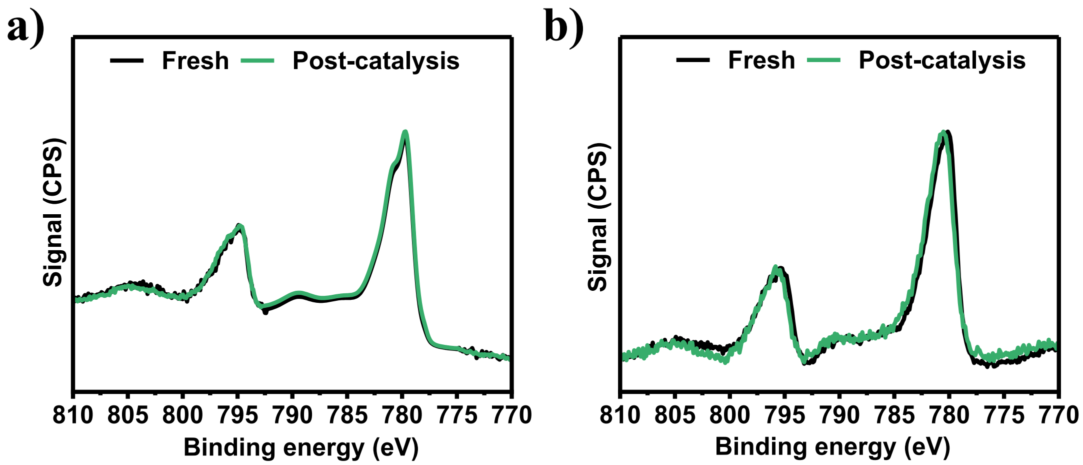

Figure S12. XPS spectra of the Co 2p region before and after a) thermal catalysis, b) EGOR.

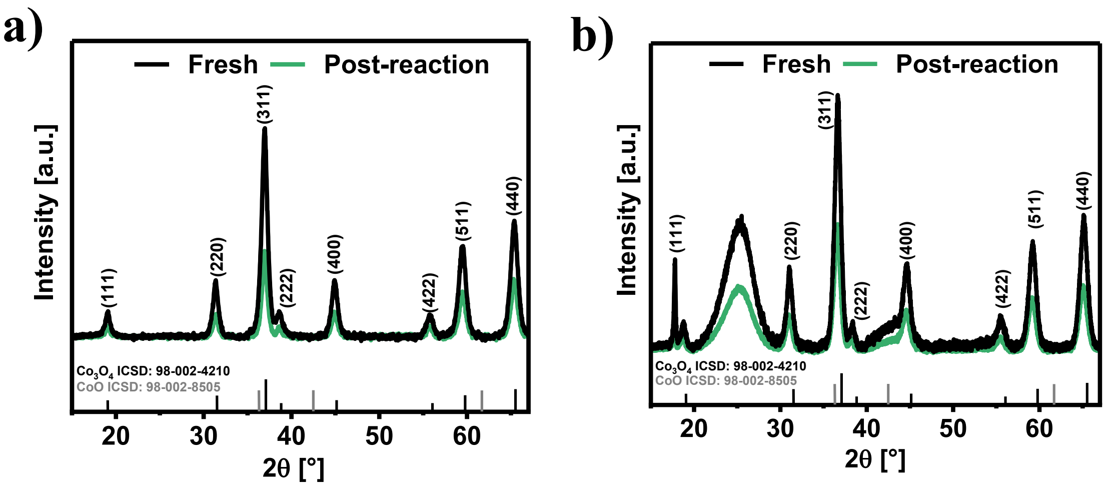

Figure S13. XRD patterns before and after the reaction of a) thermal catalysis, b) EGOR.

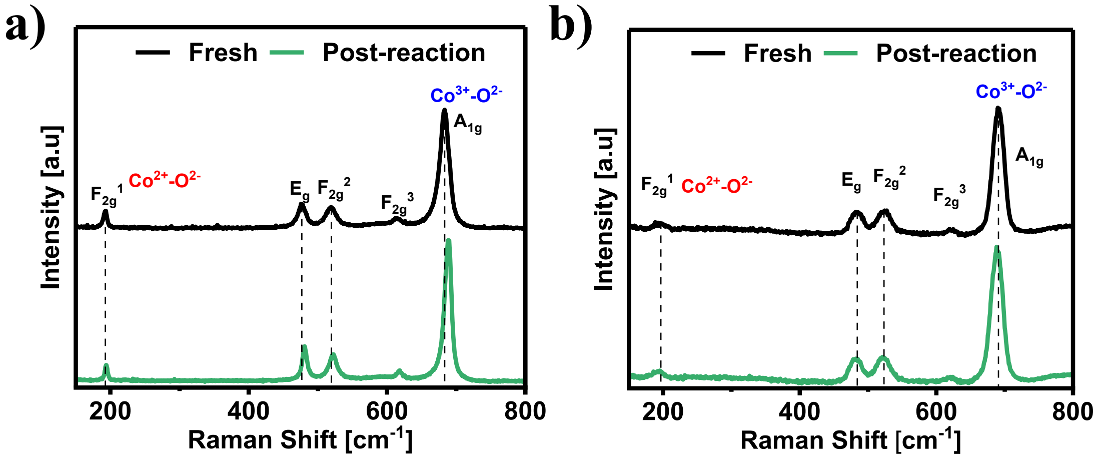

Figure S14. Raman spectra of the samples before and after reaction for a) thermal catalysis (532 nm wavelength and 300 µm confocal point hole) and b) EGOR (532 nm wavelength and 300 µm confocal point).

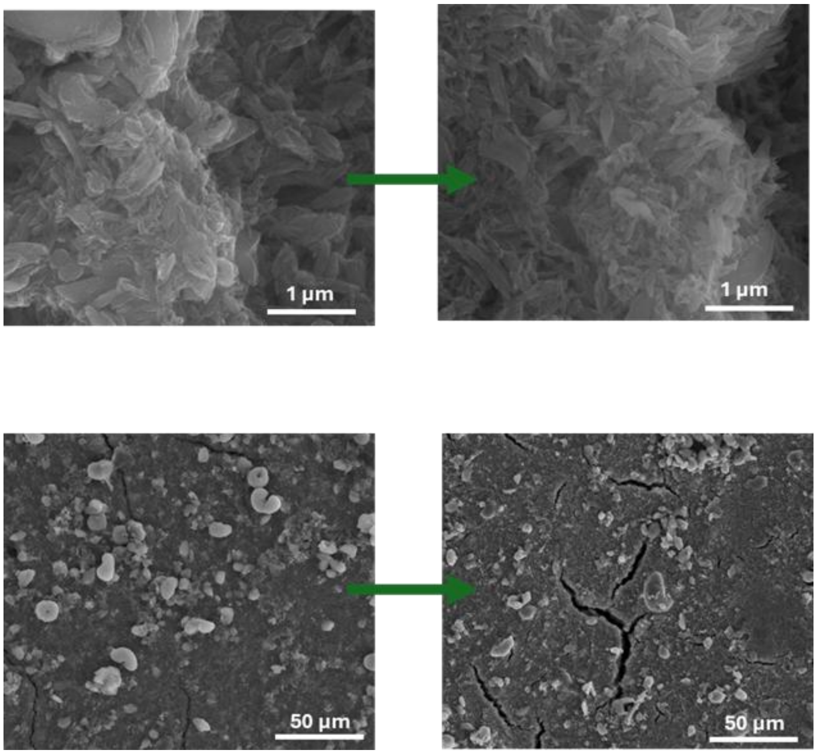

Figure S15. SEM before and after EGOR***.***

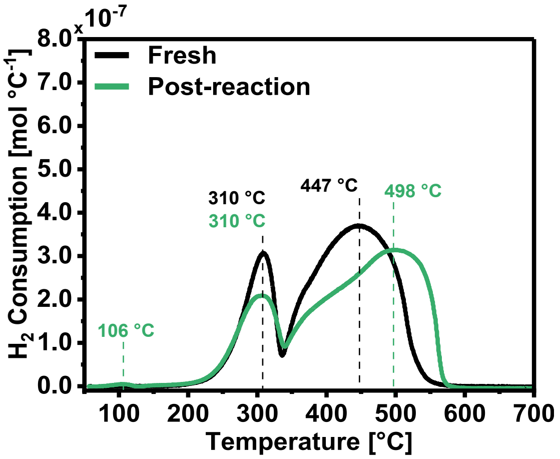

Figure S16. H_2 TPR profiles of the Co_3O_4 samples before and after thermal catalysis.

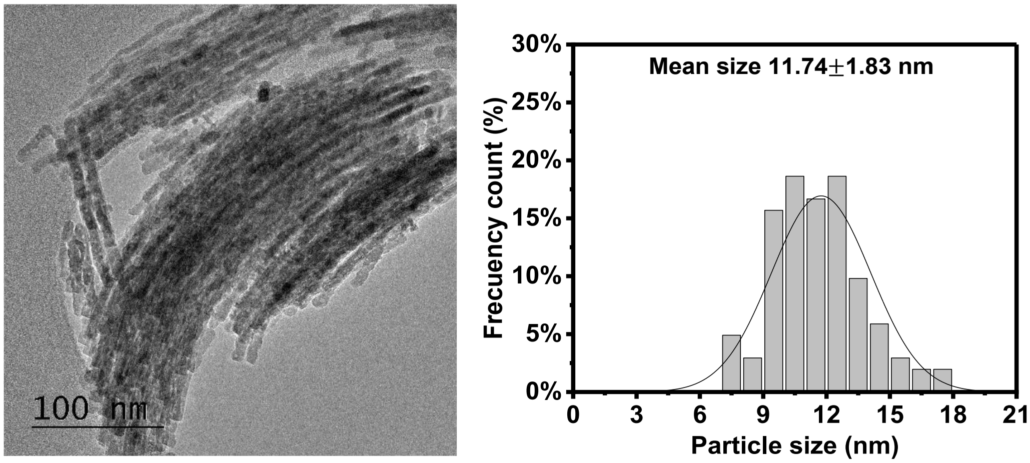

Figure S17. TEM images and particle size distribution after thermal catalysis.

Table S3. Summary of TEM and XRD results on the samples before and after thermal catalysis.

**TEM**[a]		**XRD**			
**Fresh**	**Post-reaction**	**Fresh**		**Post-reaction**	
d_P **(nm)**	d_P **(nm)**	d_P[b] **(nm)**	**Lattice parameter (Å)**	d_P[b] **(nm)**	**Lattice parameter (Å)**
10.7	11.7	8.7	8.071	10.0	8.070

[a] Calculated by the number average using 100 nanoparticles.
[b] Calculated using the Scherrer equation.

Table S4. XRD results of the samples before and after EGOR.

**Fresh**		**Post-reaction**	
***d_P*[a] (nm)**	**Lattice parameter (Å)**	***d_P*[a] (nm)**	**Lattice parameter (Å)**
7.9	8.142	8.07	8.132

[a] Calculated using the Scherrer equation.

Table S5. Hydrogen consumption of the samples after reaction during the stepwise reduction of Co_3O_4 to metallic Co^0.

	**$Co^{3+} \rightarrow Co^{2+}$**	**$Co^{2+} \rightarrow Co^0$**	
**Mass (mg)**	**1st H_2 consumption (mmol)**	**2nd H_2 consumption (mmol)**	$\frac{Co^{3+}}{Co^{2+}}$
58.5	0.15	0.53	1.7

Computational details

Spin-polarized Born–Oppenheimer molecular dynamics (BOMD) simulations were performed using the CP2K/Quickstep package[15] at the point and considering the generalized gradient approximation (GGA) within its PBE functional.[16] To account for the self-interaction in the cobalt 3d orbitals, a Hubbard U correction of 2 eV was applied, as described in our previous work.[17-20] Valence electrons (2s and 2p for oxygen; 3s, 3p, 3d, and 4s for Co^{2+} and Co^{3+}) were treated using GTH-type pseudopotentials[21] with a kinetic energy cut-off of 500 Ry, and Gaussian-type local basis sets were employed for the electronic wave functions.[22] Simulations were carried out under NVT conditions, with a Nosé–Hoover thermostat using a 0.5 fs timestep and a 100 fs time constant. A temperature of 120 °C was used to simulate aerobic thermal oxidation, while electrochemical oxidation was investigated at room temperature. Low Miller index surfaces (001) and (110) of Co_3O_4 were considered. Orthorhombic (2 × 2) supercells with x, y, z dimensions of 16.18 × 16.18 × 30 Å^3 for the (001) surface, 22.88 × 16.18 × 26.29 Å^3 for were used to study the (001) and (110) surfaces. Water films containing 140 and 184 molecules simulated the aqueous solvent. The top and bottom layers of the slabs were allowed to relax together with the electrolyte, while the middle layers were fixed at their bulk positions. Precisely, 3 out of 13 and 2 out of 8 atomic layers in the middle of (001) and (110) surface slabs, respectively. All systems were initially equilibrated for at least 2 ps, and 20-ps production runs were further performed.

Results

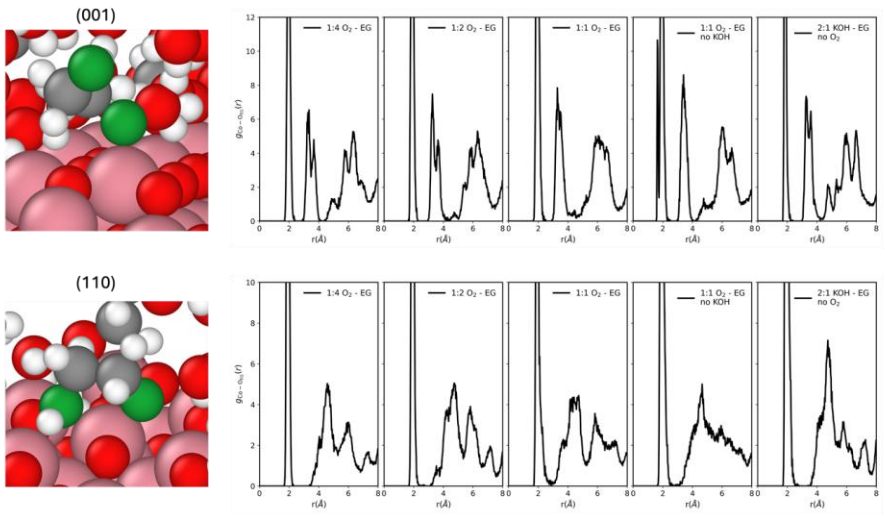

Figure S18. (Left panel) EG binding motifs on the (001, top) and (110, bottom) surfaces. Co^{3+} is pink, and surface and water oxygen is red. The atoms of EG are coloured: oxygen in green, hydrogen in white, and carbon in grey. For the sake of clarity, not all water molecules are displayed. (Right panel) Radial distribution functions (RDFs) of cobalt to EG's oxygen and surface oxygen to EG's hydrogen for different concentrations of O_2, with and without alkaline conditions. The RDFs show the interfacial bond lengths between surface Co and O_{EG}, indicating strong covalent bonding at the interface (peaks at 2 Å).

Table S6. Dissociation degree of water molecules (in %) on the (001) and (110) surfaces for various O_2 concentrations under neutral and alkaline conditions.

Oxidizing agents		Dissociation degree	
n KOH	n O_2	(001)	(110)
8	0	47.2	66.2
8	1	56.5	62.8
8	2	51.2	57.3
8	4	70.7	50.6
0	4	64.6	72.6

Interfacial molecular water and hydroxyls sit epitaxially (Figure S18) and almost in the same plane, as supported by the location of their O peaks (Figure S19). Meanwhile, their H distribution shows peaks at the same positions as the O counterparts and on the left- and right-hand sides of the latter, indicating corrugated interfacial water layers with H pointing towards the surface within the surface plane and the first solvation shells of bulk water. The interfacial roughness is more pronounced on the (110) surface as the distribution of interfacial H shows mainly distinct peaks on either side of the O peaks. Besides, the OH peaks are more pronounced, indicating a higher dissociation degree of interfacial water than the (001) surface in most cases, as shown in Table S6. However, on both surfaces, this dissociation degree is considerable and increases with the O_2 vapour pressure, which supports the increased reactivity of EG as the availability of proton acceptors increases.

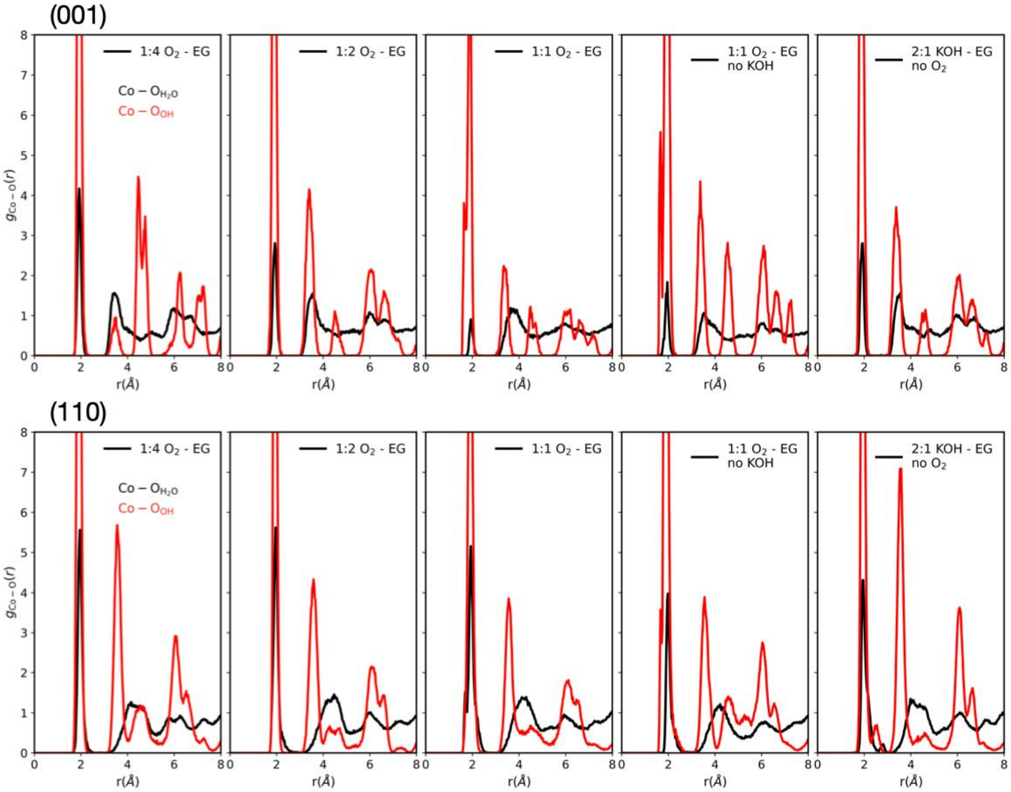

Figure S19. Radial distribution function (RDF) of cobalt to water oxygen at different O_2 concentrations under neutral and alkaline conditions.

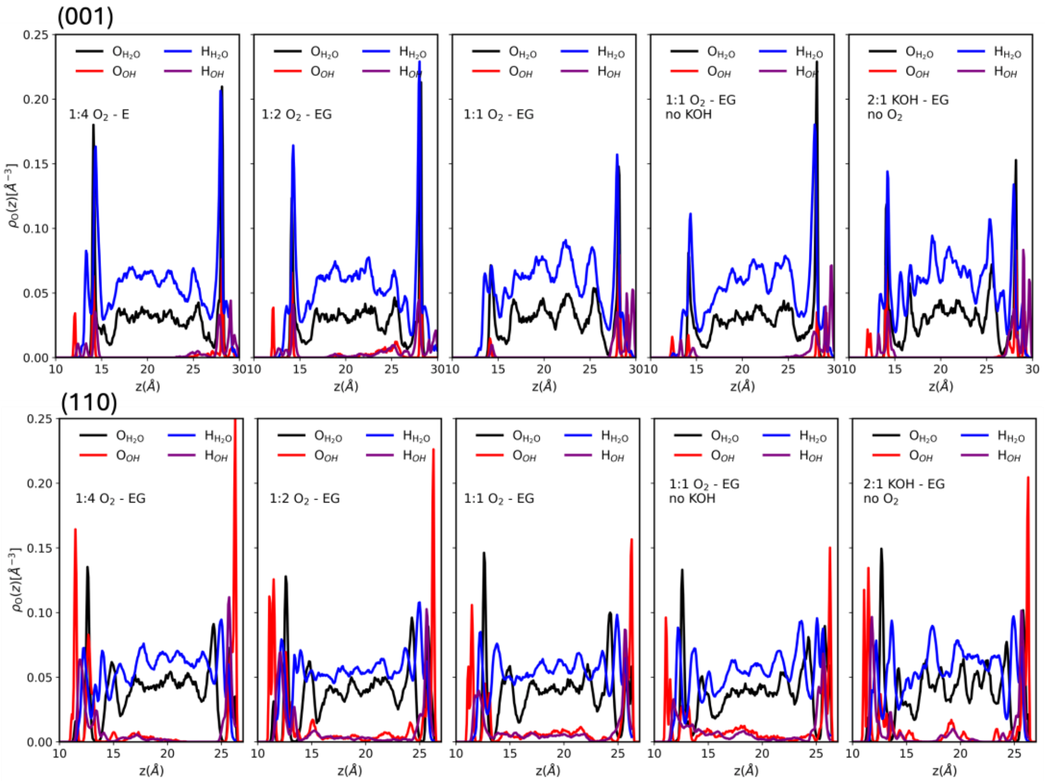

Figure S20. Average density profiles of water and hydroxyl's oxygen and hydrogen atoms at different O_2 concentrations under neutral and alkaline conditions.

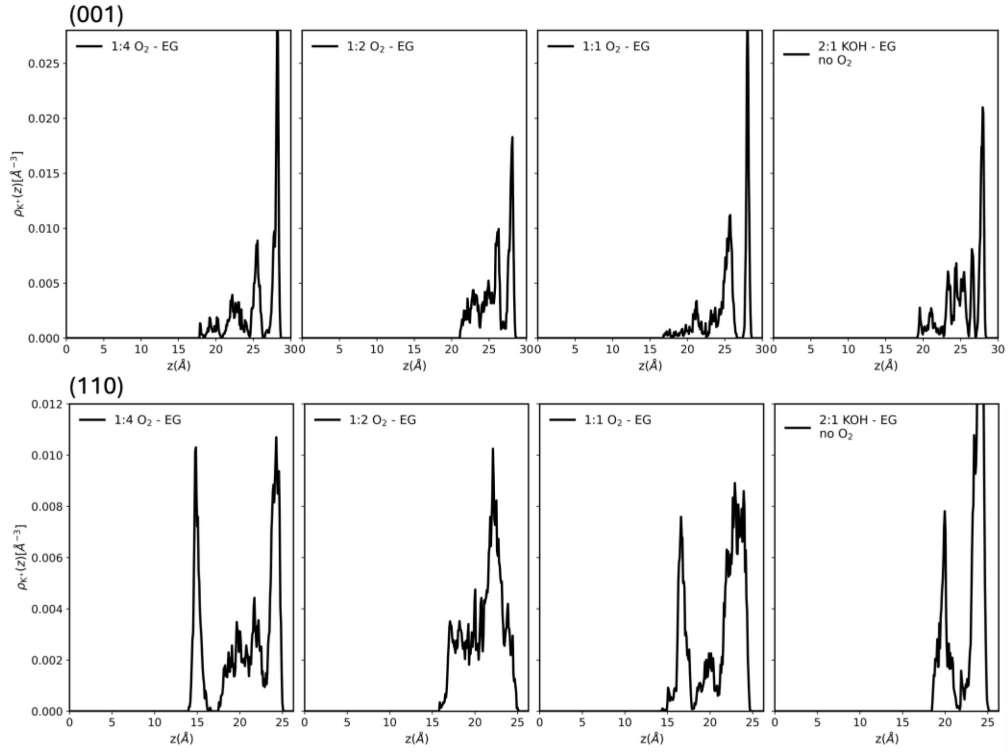

Figure S21. Density profile of K^+ vs. O_2 concentration under neutral and alkaline conditions.

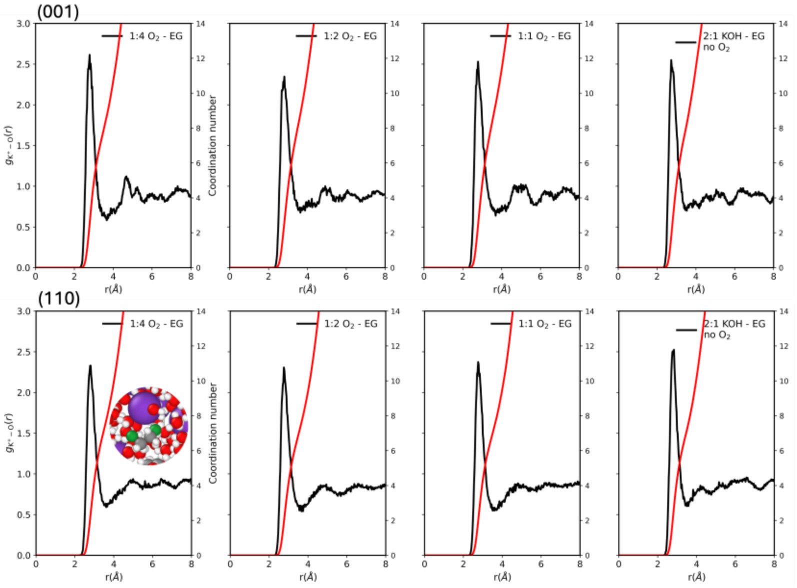

Figure S21. Radial distribution functions of alkali ions to water and hydroxyl oxygen and their corresponding running integrals, or different concentrations of O_2, with and without alkaline conditions

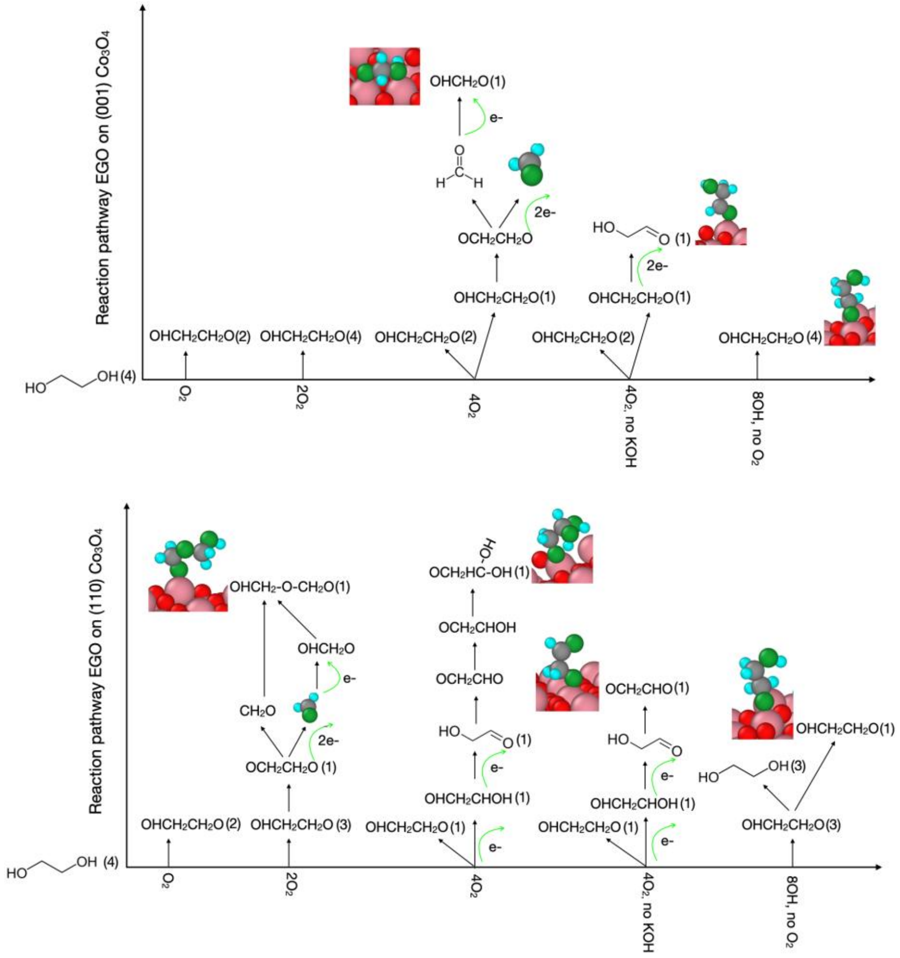

Figure S22. Reaction pathway to EG's oxidation intermediates, and products on the (001) and (110) surfaces of Co_3O_4. The numbers of intermediate and product species present in solution are given in parentheses. Metastable intermediates are not attributed a number. Cobalt atoms are represented in pink, surface oxygen depicted in red, carbon in gray, glycol's oxygens in green, and glycol's hydrogens in cyan.

Figure S22 shows the time distribution of intramolecular bond lengths in the $OHCH_2$–O–CH_2O intermediate observed on the (001) and (110) surfaces. On the (110), (middle inset, bottom), C–C bond cleavage occurs around 2 ps (black curve) already at 1:2 O_2-EG ratio, as indicated by the steep increase in the bond distance. This yields the formation of two formaldehyde (FA) groups. One FA group captures an OH^- from the electrolyte at 5 ps (see the decrease in the green curve) and further recombines with the second FA to form an epoxide ring, as indicated by a shift down in the orange curve. Meanwhile, the C=O double bonds in each FA moiety elongate with single-bond character (blue and red curves).

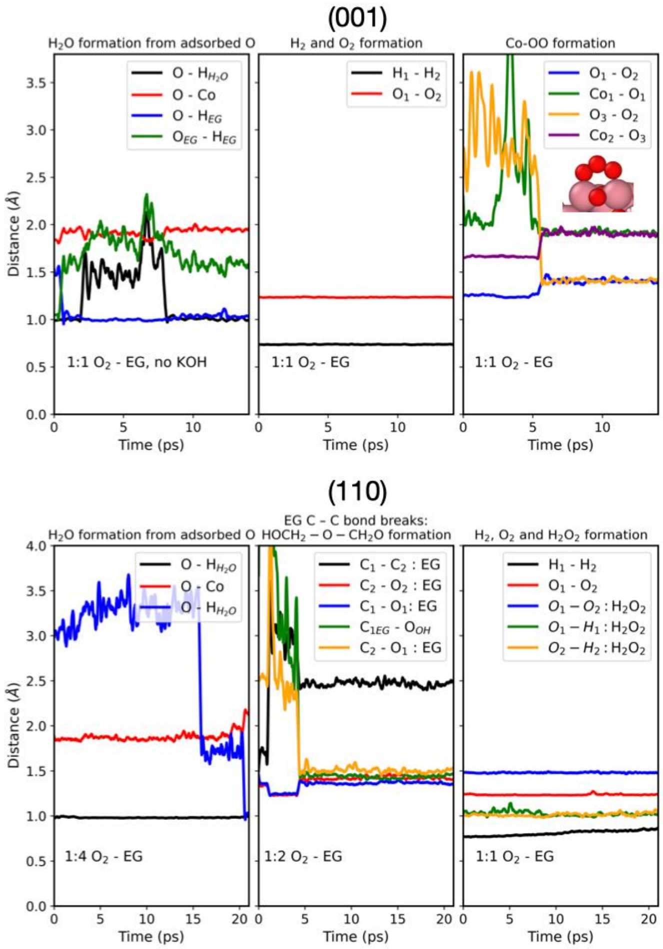

Figure S23. Time evolution of characteristic bond distances between reactive EG molecules, adsorbed atomic oxygen, and water for selected cases.

The surface-assisted EG's decomposition occurs prominently via the Langmuir–Hinshelwood (LH) mechanism. Such a mechanism occurring on the (001) surface in alkaline-free conditions is illustrated in Figure S23 (top left). An adsorbed oxygen atom abstracts a hydrogen atom from ethylene glycol (blue curve), as evidenced by a decrease in the O–H_{EG} bond distance and a parallel increase in the O_{EG}–H_{EG} distance (green curve), which leads to the formation of a hydroxyl adsorbate. The latter subsequently abstracts a hydrogen atom from a surrounding water molecule (O–H_{H_2O}, black curve). The resulting water molecule forms at approximately 8 ps, as supported by increased bond length between the Co site on which hydroxyl was adsorbed, now present as molecular water (O–Co, red curve). This O-Co bond length increases from 1.8 to ~2 Å. The LH mechanism is also prominent on the (110) surface as illustrated in Figure S18 (bottom left). An oxygen adsorbate abstracts two hydrogen atoms from the electrolyte (black and blue curves) to form a water molecule. The corresponding Co–O bond distance increases progressively from ~1.7 Å (cobalt-oxo state) to ~2.0 Å after 16 ps, indicating the weakening of the interaction with the surface due to bonding to molecular water.

Figure S23 (top middle) supports the stability of H-H and O-O bonds and the sustainability of molecular H_2 (black curves), O_2 (red curves) evolution in alkaline conditions. Figure S23 (bottom right) also exteriorises the formation and stability of H_2O_2. These observations also hold in alkaline-free medium and under anodic conditions. Both O_2 and H_2O_2 can serve as reoxidizing agents of surface cobalt species. For example, as shown in Figure S23 (top right), O_2 adsorbates can arise from the bonding between an oxygen adsorbate and an oxygen from the electrolyte. The resulting O–O bond (O_1-O_2, blue curve) shows a single-bond character with a corresponding bond length of 1.2 Å. This yields Co–OO species formation after stabilizing the adjacent Co-O bond (Co– O_1 bond, green curve). At approximately 5 ps, the resulting cobalt oxyhydroxide further interacts with an oxygen adsorbate (O_2 – O_3, orange curve), strongly bonded to a cobalt site (Co_2–O_3 bond, purple curve) in a cobalt-oxo state. This yields the elongation of the Co_2–O_3 bond (~1.6 to 2 Å) and the formation of a metastable Co–OOO complex.

The electrolyte pH at different O_2 concentrations for both (001) and (110) surfaces was calculated using the following approach: the number of solvate OH^- ions originating from the dissociation of KOH was used to calculate the molar concentration of OH^- in the electrolyte. From this concentration, the pOH value was obtained, from which the pH can be directly derived as follows:

$pH = pKw – pOH$ with $pOH= -\log [OH^-]$.

The pKw of water was taken as 14 at 25 °C and 12 at 120 °C.

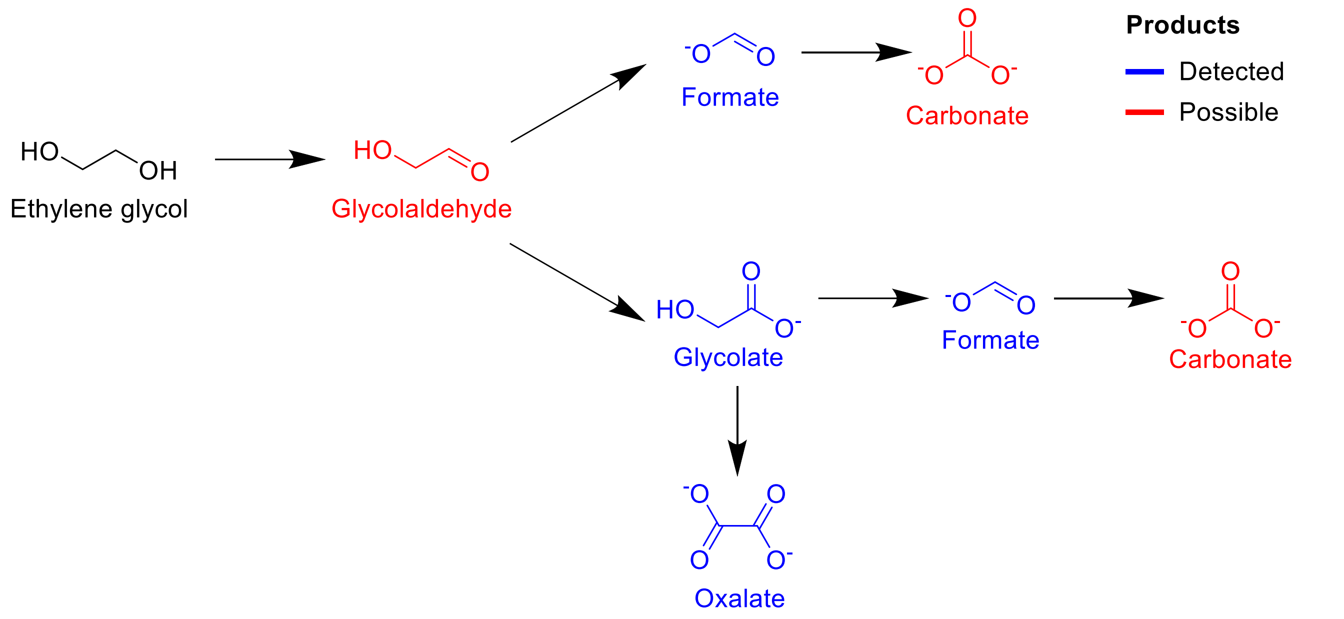

Scheme S1. Reaction network of the ethylene glycol oxidation, including detected products (formate, glycolate and oxalate) and possible products (glycolaldehyde and carbonate).

Table S7. Calculated pH at different concentrations of O_2 under alkaline conditions. The number of dissociated OH^- is shown in parentheses.

Oxidizing agents		pH values	
n KOH	n O_2	(001)	(110)
8	0	13.6 (1)	14.1 (4)
8	1	12.1 (3)	12.2 (5)
8	2	11.6 (1)	12.3 (6)
8	4	12.2 (4)	12.2 (5)

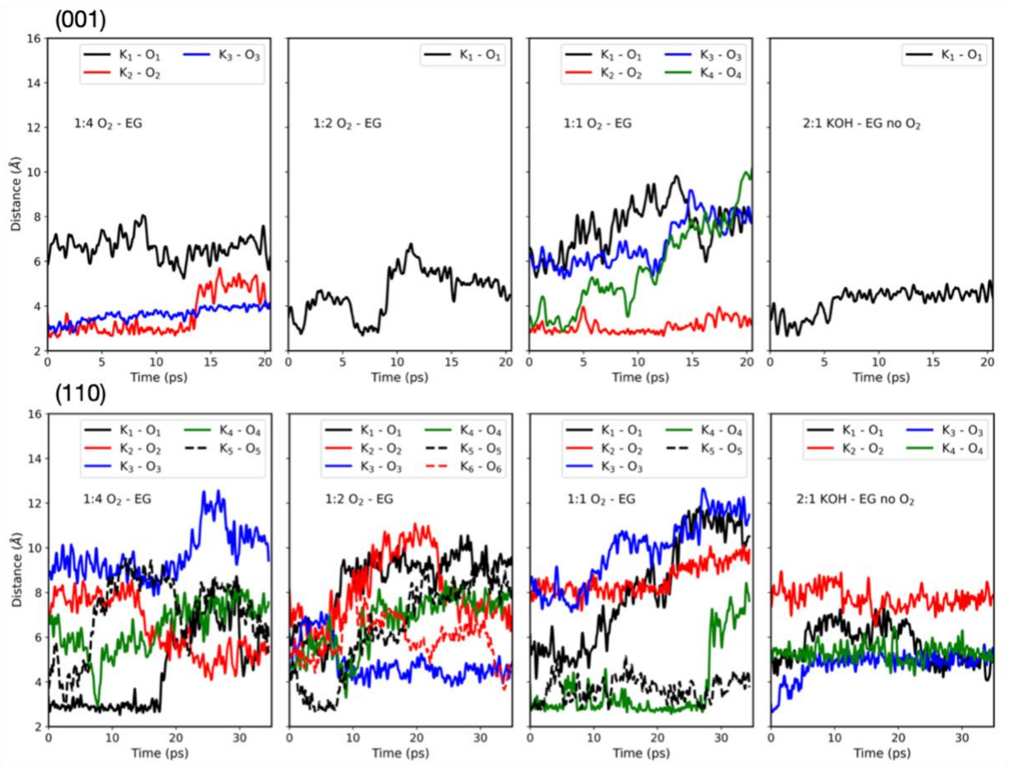

Figure S24. Time evolution of the bond distances between dissociated OH^- ions and their corresponding K^+ ions.

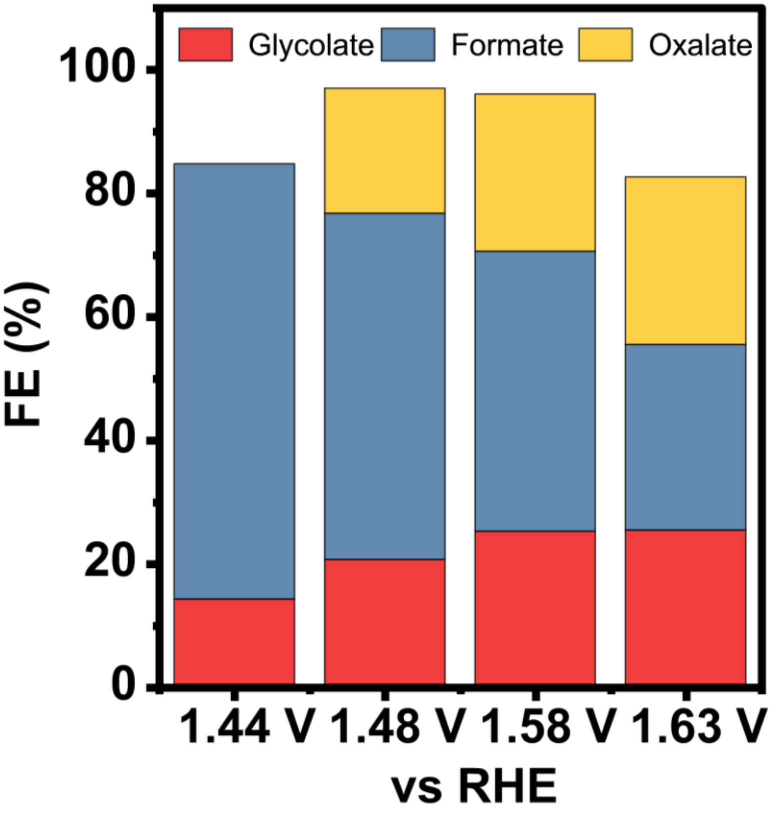

Figure S26. Faradaic efficiency values for potential dependant measurements done at 1 M KOH and 1 M EG.

Faradaic efficiency (FE) analysis was carried out under potential-dependent conditions. Formate exhibited the highest FE, followed by glycolate and oxalate, consistent with the observed product selectivity. At

higher applied potentials, the FE of all products decreased, likely due to further oxidation towards carbonate. The total FE values were slightly below 100%, which can be attributed to parasitic OER and partial conversion of intermediates into carbonates.

REFERENCES

[1] X. Deng, K. Chen, H. Tüysüz, "Protocol for the nanocasting method: Preparation of ordered mesoporous metal oxides", *Chem. Mater.* **2016**, *29*, 40-52.

[2] M. Dreyer, A. Rabe, E. Budiyanto, K. Friedel Ortega, S. Najafishirtari, H. Tüysüz, M. Behrens, "Dynamics of reactive oxygen species on cobalt-containing spinel oxides in cyclic CO oxidation", *Catalysts* **2021**, *11*, 1312.

[3] E. Budiyanto, M. Yu, M. Chen, S. DeBeer, O. Rüdiger, H. Tüysüz, "Tailoring morphology and electronic structure of cobalt iron oxide nanowires for electrochemical oxygen evolution reaction", *ACS Appl. Energy Mater.* **2020**, *3*, 8583-8594.

[4] R. Al-Tuwirqi, A. A. Al-Ghamdi, N. A. Aal, A. Umar, W. E. Mahmoud, "Facile synthesis and optical properties of Co_3O_4 nanostructures by the microwave route", *Superlatt. Microstruct.* **2011**, *49*, 416-421.

[5] D. Waffel, E. Budiyanto, T. Porske, J. Büker, T. Falk, Q. Fu, S. Schmidt, H. Tüysüz, M. Muhler, B. Peng, "Investigation of synergistic effects between Co and Fe in $Co_{3-x}Fe_xO_4$ spinel catalysts for the liquid-phase oxidation of aromatic alcohols and styrene", *Mol. Catal.* **2020**, *498*, 111251.

[6] L. Hu, Q. Peng, Y. Li, "Selective synthesis of Co_3O_4 nanocrystal with different shape and crystal plane effect on catalytic property for methane combustion", *J. Am. Chem. Soc.* **2008**, *130*, 16136-16137.

[7] F. T. Haase, E. Ortega, S. Saddeler, F. P. Schmidt, D. Cruz, F. Scholten, M. Ruscher, A. Martini, H. S. Jeon, A. Herzog, U. Hejral, E. M. Davis, J. Timoshenko, A. Knop-Gericke, T. Lunkenbein, S. Schulz, A. Bergmann, B. Roldan Cuenya, "Role of Fe decoration on the oxygen evolving state of Co_3O_4 nanocatalysts", *Energy Environ. Sci.* **2024**, *17*, 2046-2058.

[8] D. Adekoya, H. Chen, H. Y. Hoh, T. Gould, M. J. T. Balogun, C. Lai, H. Zhao, S. Zhang, "Hierarchical Co_3O_4@N-doped carbon composite as an advanced anode material for ultrastable potassium storage", *ACS Nano* **2020**, *14*, 5027-5035.

[9] C.-W. Tang, C.-B. Wang, S.-H. Chien, "Characterization of cobalt oxides studied by FT-IR, Raman, TPR and TG-MS", *Thermochim. Acta* **2008**, *473*, 68-73.

[10] T. Falk, E. Budiyanto, M. Dreyer, J. Büker, C. Weidenthaler, M. Behrens, H. Tüysüz, M. Muhler, B. Peng, "Doping of nanostructured Co_3O_4 with Cr, Mn, Fe, Ni, and Cu for the selective oxidation of 2-propanol", *ACS Appl. Nano Mater.* **2022**, *5*, 17783-17794.

[11] E. van Steen, G. S. Sewell, R. A. Makhothe, C. Micklethwaite, H. Manstein, M. de Lange, C. T. O'Connor, "TPR study on the preparation of impregnated Co/SiO_2catalysts", *J. Catal.* **1996**, *162*, 220-229.

[12] J.-b. Li, Z.-q. Jiang, K. Qian, W.-x. Huang, "Effect of calcination temperature on surface oxygen vacancies and catalytic performance towards CO oxidation of Co_3O_4 nanoparticles supported on SiO_2", *Chin. J. Chem. Phys.* **2012**, *25*, 103.

[13] V. Hadjiev, M. Iliev, I. Vergilov, "The raman spectra of Co_3O_4", *J. Phys. C: Solid State Phys.* **1988**, *21*, L199.

[14] S. R. Gawali, A. C. Gandhi, S. S. Gaikwad, J. Pant, T. S. Chan, C. L. Cheng, Y. R. Ma, S. Y. Wu, "Role of cobalt cations in short range antiferromagnetic Co_3O_4 nanoparticles: a thermal treatment approach to affecting phonon and magnetic properties", *Sci. Rep.* **2018**, *8*, 249.

[15] T. D. Kuhne, M. Iannuzzi, M. Del Ben, V. V. Rybkin, P. Seewald, F. Stein, T. Laino, R. Z. Khaliullin, O. Schutt, F. Schiffmann, D. Golze, J. Wilhelm, S. Chulkov, M. H. Bani-Hashemian, V. Weber, U. Borstnik, M. Taillefumier, A. S. Jakobovits, A. Lazzaro, H. Pabst, T. Muller, R. Schade, M. Guidon, S. Andermatt, N. Holmberg, G. K. Schenter, A. Hehn, A. Bussy, F. Belleflamme, G. Tabacchi, A. Gloss, M. Lass, I. Bethune, C. J. Mundy, C. Plessl, M. Watkins, J. VandeVondele, M. Krack, J. Hutter, "CP2K: An electronic structure and molecular dynamics software package - Quickstep: Efficient and accurate electronic structure calculations", *J. Chem. Phys.* **2020**, *152*, 194103.

[16] J. P. Perdew, K. Burke, M. Ernzerhof, "Generalized gradient approximation made simple", *Phys. Rev. Lett.* **1996**, *77*, 3865-3868.
[17] A. H. Omranpoor, S. Kenmoe, "2-Propanol activation on the low index Co_3O_4 surfaces: A comparative study using molecular dynamics simulations", *Catalysts* **2023**, *14*, 25.
[18] F. B. S. Nkou, S. Kenmoe, "Ethylene glycol partial oxidation on Co_3O_4 (001) surface: Interplay between solute's surface coverage and aqueous solvation", *Mol. Catal.* **2025**, *578*, 114980.
[19] F. B. S. Nkou, S. Kenmoe, "Ethylene glycol partial aqueous oxidation on Co_3O_4 (001) surfaces: pathways to two- and four-electron products in neutral and oxidative conditions", *ChemCatChem* **2025**, *17*, e202401885.
[20] T. Kox, S. Kenmoe, "Co_3O_4 (111) surfaces in contact with water: molecular dynamics study of the surface chemistry and structure at room temperature", *Dalton Trans.* **2024**, *53*, 13184-13194.
[21] S. Goedecker, M. Teter, J. Hutter, "Separable dual-space Gaussian pseudopotentials", *Phys. Rev. B* **1996**, *54*, 1703-1710.
[22] G. Lippert, J. Hutter, M. Parrinello, "The Gaussian and augmented-plane-wave density functional method for ab initio molecular dynamics simulations", *Theor. Chem. Acc.* **1999**, *103*, 124-140.
